# Development of a Novel Passive Monitoring Technique to Showcase the 3D Distribution of Tritiated Water (HTO) Vapor in Indoor Air of a Nuclear Facility

**DOI:** 10.1021/acs.est.3c05783

**Published:** 2023-11-15

**Authors:** Bin Feng, Martin Ibesich, Dieter Hainz, Daniel Waidhofer, Monika Veit-Öller, Clemens Trunner, Thomas Stummer, Michaela Foster, Markus Nemetz, Jan M. Welch, Mario Villa, Johannes H. Sterba, Andreas Musilek, Franz Renz, Georg Steinhauser

**Affiliations:** † Institute of Applied Synthetic Chemistry & TRIGA Center Atominstitut, TU Wien, 1060 Vienna, Austria; ‡ Institute of Inorganic Chemistry, Leibniz Universität Hannover, 30167 Hannover, Germany; § TRIGA Center Atominstitut, TU Wien, 1020 Vienna, Austria

**Keywords:** tritium, passive sampler, indoor air pollution, nuclear industry, 3D spatial distribution, environmental radioactivity

## Abstract

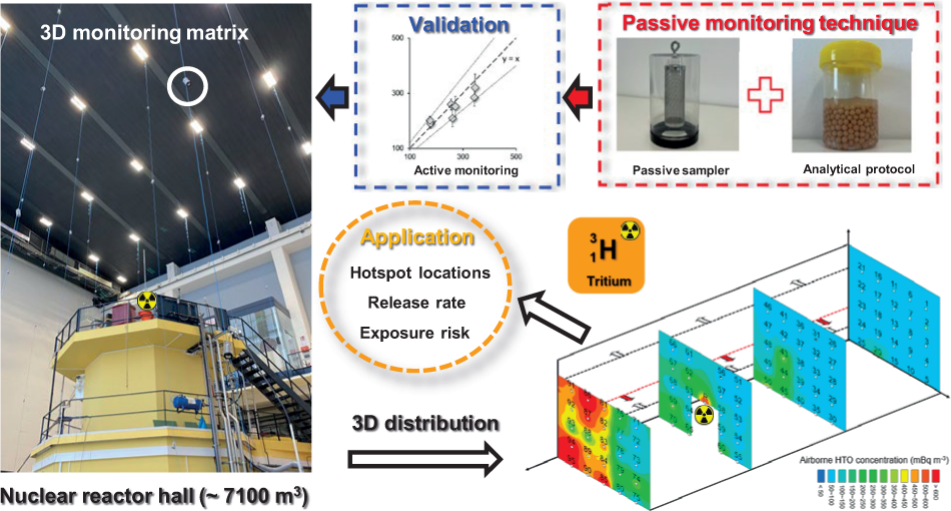

Tritiated water (HTO), a ubiquitous byproduct of the nuclear industry, is a radioactive contaminant of major concern for environmental authorities. Although understanding spatiotemporal heterogeneity of airborne HTO vapor holds great importance for radiological safety as well as diagnosing a reactor’s status, comprehensive HTO distribution dynamics inside nuclear facilities has not been studied routinely yet due to a lack of appropriate monitoring techniques. For current systems, it is difficult to simultaneously achieve high representativeness, sensitivity, and spatial resolution. Here, we developed a passive monitoring scheme, including a newly designed passive sampler and a tailored analytical protocol for the first comprehensive 3D distribution characterization of HTO inside a nuclear reactor facility. The technique enables linear sampling in any environment at a one-day resolution and simultaneous preparation of hundreds of samples within 1 day. Validation experiments confirmed the method’s good metrological properties and sensitivity to the HTO’s spatial dynamics. The air in TU Wien’s reactor hall exhibits a range of ^3^H concentrations from 75–946 mBq m^–3^ in the entire 3D matrix. The HTO release rate estimated by the mass-balance model (3199 ± 306 Bq h^–1^) matches the theoretical calculation (2947 ± 254 Bq h^–1^), suggesting evaporation as the dominant HTO source in the hall. The proposed method provides reliable and quality-controlled 3D monitoring at low cost, which can be adopted not only for HTO and may also inspire monitoring schemes of other indoor pollutants.

## Abstract 2

A novel passive monitoring technique was developed, validated, and applied to characterize the 3D spatiotemporal heterogeneity of airborne HTO in a nuclear facility.

## Introduction

Releases of radionuclides from nuclear facilities may cause negative health effects but are even more likely to trigger public concern and hence cause socioeconomic damage.^1,2^ Comprehensive and reliable monitoring of anthropogenic radionuclides in the environment, therefore, is essential for nuclear safety and risk assessment of occupational exposure.^3−6^ Among various anthropogenic radionuclides, tritium (^3^H) is noteworthy due to its relatively long half-life (*T*_1/2_ = 12.33 years) and high migration capacity.^7−9^ As a radioactive isotope of hydrogen, ^3^H is omnipresent as tritiated water (HTO) vapor in air,^10^ leading to widespread distribution through the water cycle and food chain.^11−13^ Despite analytical challenges due to tritium’s volatility and low-energy beta decay, radiation regulatory authorities have extensively documented airborne HTO dynamics in many countries over the past decades.^14−17^ Concerning the indoor atmosphere of nuclear facilities, HTO monitoring primarily serves for surveillance monitoring to diagnose reactor status and assess occupational exposure.^18−20^ Although online tritium monitoring instruments (e.g., ionization chambers) provide timely information in case of accidental leakage,^21^ the high instrument costs, high detection limits, and single-point radiation measurement limit their ability to characterize the spatiotemporal heterogeneity of HTO in large volumes of indoor air, such as reactor halls.^22,23^ Without exact and quantitative knowledge on HTO in air, small leakages from nuclear installations cannot be pinpointed accurately, and the development of proper strategies for nuclear facility decommissioning may also influenced. Therefore, a supplementary method for the comprehensive characterization of HTO vapor in nuclear facilities is desired to meet future challenges resulting from nuclear power expansion based on both fission and fusion.

A passive ^3^H monitoring technique integrating passive sampling with liquid scintillation counting (LSC) provides a low-cost and convenient tool for quantifying airborne HTO with a high spatial resolution.^24,25^ So far, this technique has been increasingly employed in outdoor HTO monitoring scenarios.^26−32^ However, its application in indoor environments facing high tritium contamination (e.g., nuclear reactor halls) remains far from routine,^33,34^ though this concept was proposed in the 1980s.^35^ Specifically, previous studies rarely considered the variations in the adsorbent’s sampling rate after long-term exposure, in which nonlinear sampling would significantly weaken the environmental representativeness of passively collected samples.^36^ Although a recent study suggests modifying the configurations of an HTO sampler to enhance sampling representativeness, the proposed design appears too “bulky” for a flexible and convenient monitoring campaign in an indoor environment.^27^ Ideally, sampler designs should be adaptable to environments with variable humidity and allow for the monitoring of comprehensive HTO dynamics in three dimensions (3D); however, established protocols provide a 2D profile on a horizontal plane.^37,38^ For the sample preparation, the desorption method is considered time-consuming and labor-intensive, and it may also suffer from cross-contamination in the case of hundreds of contaminated samples awaiting preparation. Leaching ^3^H-contaminated materials is a convenient approach widely utilized in nuclear decommissioning;^39−41^ however, it has infrequently been considered in passive HTO monitoring frameworks due to a lack of tailored analytical protocols. Because of the above problems, the three-dimensional (3D) spatial distribution and dynamics of HTO in large reactor halls still remain unclear.

In light of these gaps, we propose a novel passive monitoring scheme, including a new HTO sampler design and a tailored leaching-based protocol, for the straightforward characterization of indoor HTO levels and thus provide fundamental information for assessment and control of HTO contamination. The sampling performance and radiometric properties of the leaching method were systematically evaluated, and the feasibility and sensitivity of the passive monitors were validated in real-life scenarios. Finally, this technique was successfully applied in a large reactor hall (∼7100 m^3^) to showcase the 3D distribution of indoor HTO, and the derived data were subsequently used in pinpointing HTO hotspots, estimating daily indoor HTO release rate, and assessing occupational exposure risks. A 3D contamination mapping not only may bring new insights for the radioactivity community but also may inspire the airborne chemical pollutants community, particularly in validating modeling predictions and identifying unexpected contaminant hotspots.

## Materials and Methods

### Sampler Design

**Figure 1:**
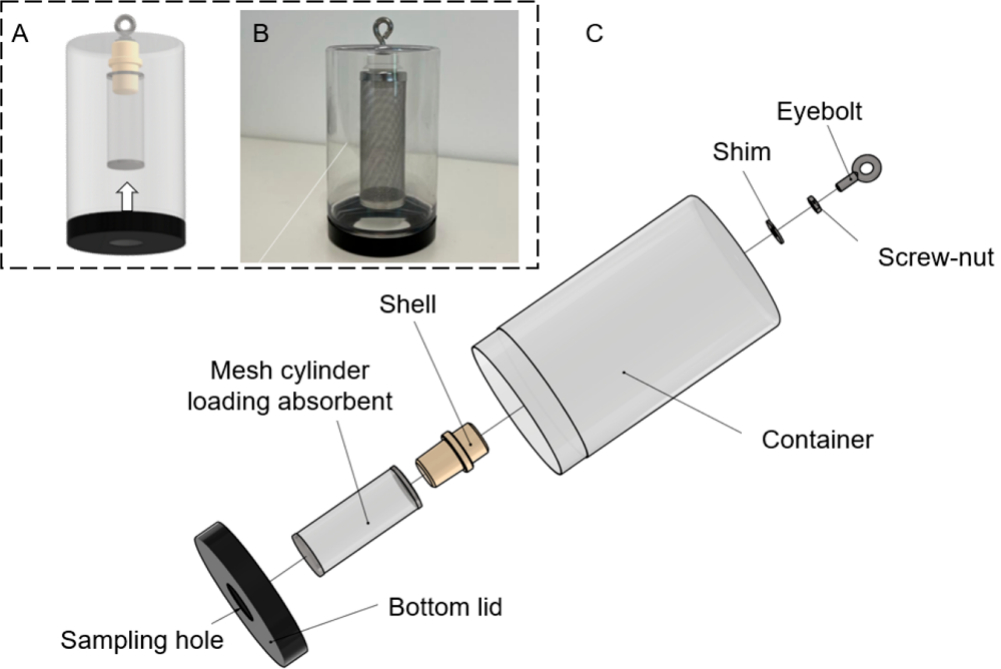
Diagrammatic sketch of the sampler design (A), sampler photo (B), and detailed structural components of the passive sampler (C).

Since indoor environments with nuclear facilities typically have less turbulence and higher tritium contents, the design concept of our passive HTO sampler was more focused on sampling representativeness, flexibility, and cost, instead of pursuing high sampling stability or ultralow detectability. Inspired by the structures of cylindrical samplers,^42,43^ an HTO passive sampler was developed using commercially available materials. As shown in Figure 1, the main sampler body is assembled with four detachable components, including a hanger, protective housing container (⌀ 71 mm × 119 mm), mesh cylinder (⌀ 30 mm × 85 mm), and a bottom lid with a sampling hole. In operation, about 30 g of zeolite 4 Å (Disidry Silicagel) was weighed into the mesh cylinder. Based on the friction forces between the shell and the opening of the mesh screen, two components can be physically connected. To avoid overadsorption of the zeolite affecting sampling representativeness during monitoring period, a modifiable bottom lid with variable diameter of the single hole was applied to adopt ambient humidity.

### Analytical Protocol for HTO

Followed by the HTO analytical framework widely used in nuclear decommissioning,^41,44^ we proposed a tailored protocol for analyzing ^3^H contents in passive sampler. Briefly, it includes three steps: (1) immersing used adsorbent into a sealable beaker loading with triple distilled water for a while; (2) separating leachate from suspended particles in leaching solution using a syringe PDVF filter (0.22 μm, ROTILABO); (3) determining ^3^H activity in the leachate by LSC. To optimize the leaching conditions, the impacts of key components, including leaching amount (10–50 mL), leaching material (zeolite 4 Å, silica gel with/without color indicator), leaching duration (1–14 days), and leaching temperature (20, 50, 80 °C), on the physicochemical properties of the leachate were systematically studied. Without specific instructions, the leaching experiments were performed in a nonradioactive laboratory at a constant temperature (20 °C). The leachate conductivity and its quenching level determined by the LSC systems (Tri-Carb 2910RT and Hidex 300SL) were used for the quantification of the influences. More details on batch experiments were given in part 1 of the Supporting Information (SI). The details of the ^3^H analysis and uncertainty assessment are provided in part 2 of the SI.

### Performance Test and Technique Validation

The calibration experiments were conducted in an unoccupied office to learn the adsorption kinetics of passive samplers. A calibrated meteorological logger (ZOGLAB) was deployed near the samplers to record the temperature and humidity dynamics concurrently with a temporal resolution of 2 min. The passive samplers were prepared as usual and equipped with variable opening bottom lids (20–60 mm) to adjust the sampling rate. Three replicate samples were used in the experiments for each sampler design, and the mass difference in the absorbent before and after sampling was measured by a calibrated balance (Kern & Sohn GmbH, 0.01 g). The adsorption kinetic curve was then established by gaining weight (g) and accumulated humidity exposure (g m^–3^ min).^27^ Therefore, by combining the average humidity (g m^–3^) back-calculated by calibrated adsorption kinetic curve with the measured ^3^H activity concentration in water vapor (Bq L^–1^), the volumetric HTO concentration in air (mBq m^–3^) can be obtained. To further test the radiometric properties (i.e., linearity, reproducibility, and minimum detectable activity) of the leaching method, the diluted ^3^H standards with various activities (∼80, 100, 500, 5000, and 10000 Bq L^–1^) were used in batch experiments (part 3 of the SI). A 7-day comparison experiment with an active sampler (Figure S1) and a passive monitoring survey that were performed in four rooms (Figure S2) with different ^3^H contamination levels were further designed for validating the feasibility and sensitivity of passive monitoring (part 4 of the SI).

### Field Application

A 3D indoor HTO monitoring matrix was constructed in the TRIGA reactor hall, where a 250 kW TRIGA Mark II reactor has been operating since 1962.^45^ Five passive samplers were fixed to a cable (∼25 m) by latches to generate a “2D sampling matrix”. Setting the position of the bottommost sampler as a horizontal reference, a constant spacing between the remaining four sampler layers was adopted to 3.5 m. Because of a slope at the roof of the reactor hall, the distance between the topmost sampler and the fixed position (lamp) was further adjusted to allow all of the top samplers to be at the same height. A total of 95 samplers were fixed on 19 sampling cables, which physically divided the reactor hall (∼7100 m^3^) into 95 submonitoring regions (Figure S3). Meanwhile, three passive samplers were deployed near the opening of the reactor pool to quantify the HTO levels at the central. After a one-day sampling, the ^3^H contents were analyzed using the established protocol. Note, the monitoring matrix was applied during the reactor shutdown due to safety considerations. Using a laser rangefinder (Bosch Professional), a coordinate system was established to describe the relative position of samplers (Figure S4). The layer-based visualizations of the spatial distribution of HTO were achieved by ArcMap v.10.8. Moreover, combining the recorded ventilation rates and investigated HTO data, the indoor HTO release rate was estimated by the mass balance model,^46^ and the value was then compared with the theoretical HTO evaporation rate from the pool port.^47−49^ The details and used parameters (Table S1) are given in part 5 of the SI.

Furthermore, the airborne HTO concentrations in the ground-floor work area were investigated during the operation. Twenty passive samplers were deployed on the workbench for a one-day sampling. Based on the typical parameters recommended by the International Commission on Radiological Protection (ICRP),^50^ the annual accumulated radiation dose (in *Sv*) induced by HTO for an adult was conservatively estimated using the investigated data during operation and shutdown (part 6 of the SI). The measures and results (Tables S2–S3, Figures S5–S6) of QA/QC are shown in part 7 of the SI.

### Statistical Analysis

The Spearman correlation analysis and Mann–Whitney test were performed using SPSS Statistics 22. The coefficient of regression analysis was obtained by a MATLAB R2020b. The measured data in the group and a single point were presented as mean ± standard deviation (*k* = 1) and mean ± combined uncertainty (*k* = 1), respectively.

## Results and Discussion

### Uptake Kinetics of a Passive Sampler

**Figure 2:**
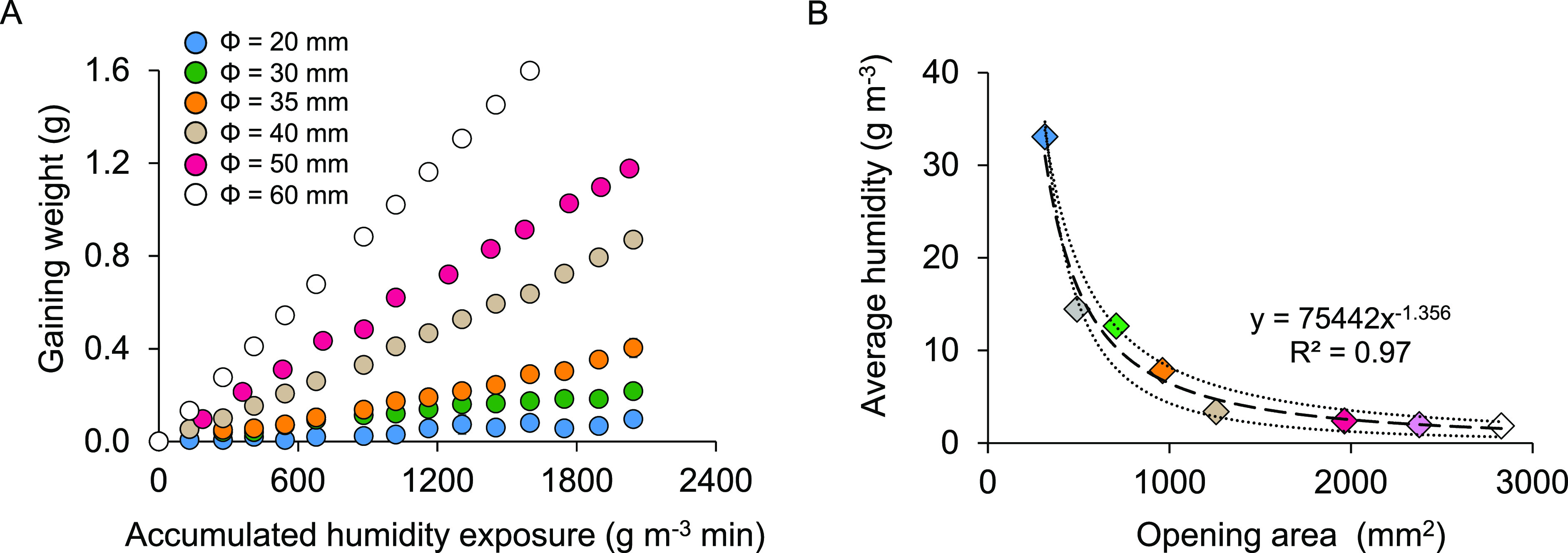
Uptake kinetics of passive samplers. (A) The adsorption kinetic curves of samplers with different opening areas. Three replicates were used for each curve, and all fitting curves were forced through zero. (B) The relationship between the opening area (mm^2^) and average humidity for a one-day sampling (g m^–3^). The dotted dashed lines are the 95% confidence intervals of the fitting curve (dash line).

By the calibration experiments, a significant difference in the sampler’s adsorption kinetic curves was observed by adjusting the opening area on the bottom lids. The sampler with larger openings reaches equilibrium much faster than smaller openings (Figure 2A). Taking 35% of the adsorbent’s equilibrium load as the end point of the linear absorption, we found that the required accumulated exposure to this threshold for a sampler with a ⌀ 20 mm opening is approximately 18-fold greater than that of a sampler with a ⌀ 60 mm opening. Further analysis demonstrates that linear sampling in the humidity range of 1.85–33.08 g m^–3^ can be achieved using samplers with an opening diameter of 20–60 mm for a one-day sampling, covering typical indoor humidity scenarios.^14^ To propose a strategy for better sampler opening modification in a specific environment, we quantitatively established the relationship (Figure 2B) between the opening area (mm^2^) and the maximum average humidity under a one-day linear sampling (g m^–3^), which thus provides a valuable tool for quickly estimating the opening size through indoor humidity. In our case, a sampler loaded with about 30 g of adsorbent can typically yield a maximum of about 2.3 g of a water sample at the end point of the linear stage. However, the uptake of the sampler with a predicted opening may sometimes exceed the linear threshold due to the inhomogeneous humidity distribution in the room. In practice, we consider a fluctuation within 20% of the linear end point still reasonable (∼2.8 g).

### Influence of Leaching Conditions on Leachate

**Figure 3:**
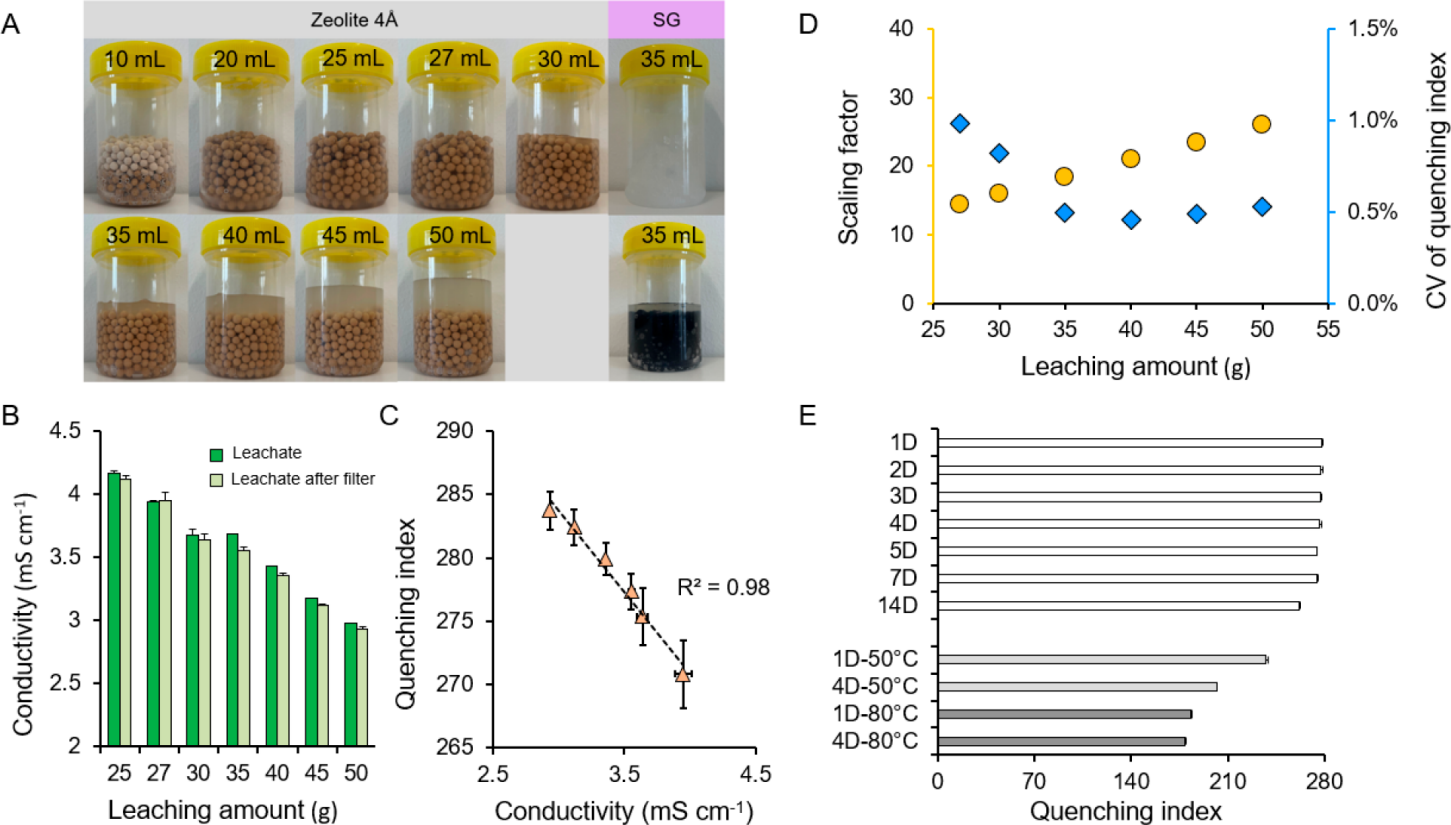
Influence of four key drivers on leaching performance. (A) Apparent effects of the leaching amount on different adsorbents. (B) Variation in conductivity of the leachate from zeolite with different leaching amounts. (C) Regression relationship (dotted line) between the conductivity and quenching index given by Tri-Carb 2910RT. (D) The influence of the added leaching amount (i.e., *m*_l_) on the scaling factor (i.e., 1 + *m*_l_/*m*_a_, the passively collected amount, *m*_a_, was set as 2 g) and the coefficient of variation (CV) of the quenching index among the replicate samples. (E) The quenching indexes of zeolite and colored silica gel in different leaching durations and temperatures. The error bars in all subfigures are the standard deviation of three replicate samples (*k* = 1).

Significant effects of leaching variables on the properties of leachate were observed in the batch experiments. By comparing the apparent states of zeolite and silica gel in the leachate (Figure 3A), it can be seen that the silica gel without a color indicator exhibits a strong swelling effect after adding water (Figure S7), while the discoloration of the orange silica gel resulted in a purple-colored leachate, which is probably due to the presence of the cobalt indicator in the silica gel. Although previous studies^41,51^ demonstrated the feasibility of analyzing ^3^H contents in leachate from trace amounts of orange silica gel (∼1 g), this material is likely not an ideal candidate in our case because a relatively high amount of silica gel with limited leaching volume may cause worse color quenching. Contrastingly, a better leachate transparency can be seen in the zeolite group after filtering the suspended matter (Figure S8). Considering the use of maximum capacity of a water sample (10 mL) with a cocktail (10 mL) to achieve the lowest-possible MDA in an LSC system would be preferable for ^3^H monitoring in an unstudied environment,^25^ a sufficient leachate with low quenching effect is thus desired, which can reflect good operability and reproducibility of the methodology. Note, although the results (Table S4) derived from the zeolite group show that 10 mL of the leachate is achievable as long as the leaching amount exceeds 25 mL, a smaller volume of the leachate would also be sufficient for ^3^H analysis in some cases with very high tritium contaminations.

Using the conductivity and quenching index, we quantified the physical properties of the zeolite leachate under different leaching conditions. Overall, the conductivity of the filtered leachate illustrates a slight decline in all groups with different leaching amounts (Figure 3B), and there is a significant decreasing trend with an increase in the leaching amount (*R*^2^ = 0.98, *P* < 0.01). Further analysis exhibits a negative correlation between the conductivity and quenching index (Figure 3C), indicating that a higher conductivity leads to a more substantial quenching degree in the leachate. Considering that the quenching effect of the leachate is related to the type and composition of the adsorbent, this phenomenon can be explained by zeolite-induced turbidity in the leachate, thus affecting the LSC system’s quenching indices. Nevertheless, a better analytical performance cannot be expected from increasing the leaching volume, as that could also result in increased dilution, and, in turn, a higher MDA (Figure 3D). In light of the leachate’s stability and detectability, 35 mL of distilled water was found appropriate for our specific purpose.

Apart from the leaching amount, the leaching duration and temperature were optimized in this study (Figure 3E). With the extension of leaching duration, the quenching index shows a slight decline trend (*r* = −0.99, *P* < 0.01) from the case of a 14-day leaching to a 1-day leaching. Moreover, at the same leaching duration, the mean leaching indices decreased with temperature significantly (*r* = −0.99, *P* < 0.01). These results support our interpolation about zeolite inducing turbidity as longer leaching duration and higher temperature promote the diffusion of suspended particles. Hence, conducting a leaching experiment at room temperature for 1 day was adopted in this work. The flowchart of the optimized leaching method is shown in Figure S9.

### Radiometric Properties of the Leaching Method

As shown in Figure S10, the ^3^H activity concentration in the leachates agreed well with the spiked ^3^H levels (*R*^2^ = 0.99, *P* < 0.01) after a 1-day leaching, but the slope of the fitting line (*Y* = 0.97 · *X*) is found slightly below 1. The remeasurement taken one and four months after sample preparation shows little changes in the slope of this curve, with a CV of about 2.7% (Figure S11), indicating the long-term stability of prepared samples. Furthermore, there is no significant increasing trend in the measured tritium content with an increasing leaching duration (Figure S12). These results are generally similar to the early finding where about 97.84% of tritium was recovered after 24 h in the leachate of silica gel, though the previous study used a higher ^3^H spike (∼2 × 10^5^ Bq L^–1^) and a higher leaching ratio (H_2_O:adsorbent = 3:1).^41^ Collectively, our results demonstrate the independence of ^3^H recovery from the original tritium contents and leaching duration. Nevertheless, a correction coefficient of 0.97 was still adopted in all measured data to make the measurement more accurate.

The MDA of the method for the LSC system was estimated by varying the counting time and adsorbent’s gaining weight. Given that the collected water vapor typically ranges from 1.5 to 2.5 g after a 1-day exposure, assuming the counting duration is 6–24 h, the MDA of approximately 30–95 Bq L^–1^ and 20–70 Bq L^–1^ thus can be achieved in Hidex 300 SL (Figure S13A) and Tri-Carb 2910RT (Figure S13B), respectively. Although the airborne ^3^H contents in nuclear facilities highly depend on the monitoring location, nuclear reactor type, and operational status, a relatively conservative threshold of 100 Bq L^–1^ is widely adopted as a “positive” event.^52^ In this context, at a hypothetical condition where gaining weight is about 1.5 g and the ^3^H content is about 100 Bq L^–1^, the relative uncertainty (*k* = 1) of approximately 8% and 15% can be obtained after 1 day of counting in Tri-Carb 2910RT and Hidex 300SL, respectively. Hence, the established leaching method is considered sensitive enough to identify ^3^H contamination in indoor environments. For a limited gaining weight (e.g., 0.7 g), a long counting duration (more than 48 h) would be preferable for satisfying measurement uncertainty. In short, the leaching method presented in this work enables rapid sample preparation and accurate determination of ^3^H hotspots, thus promoting our ability to identify those locations inside the facility whose HTO levels deviate from this “normal range”.

### Technique Evaluation

**Figure 4:**
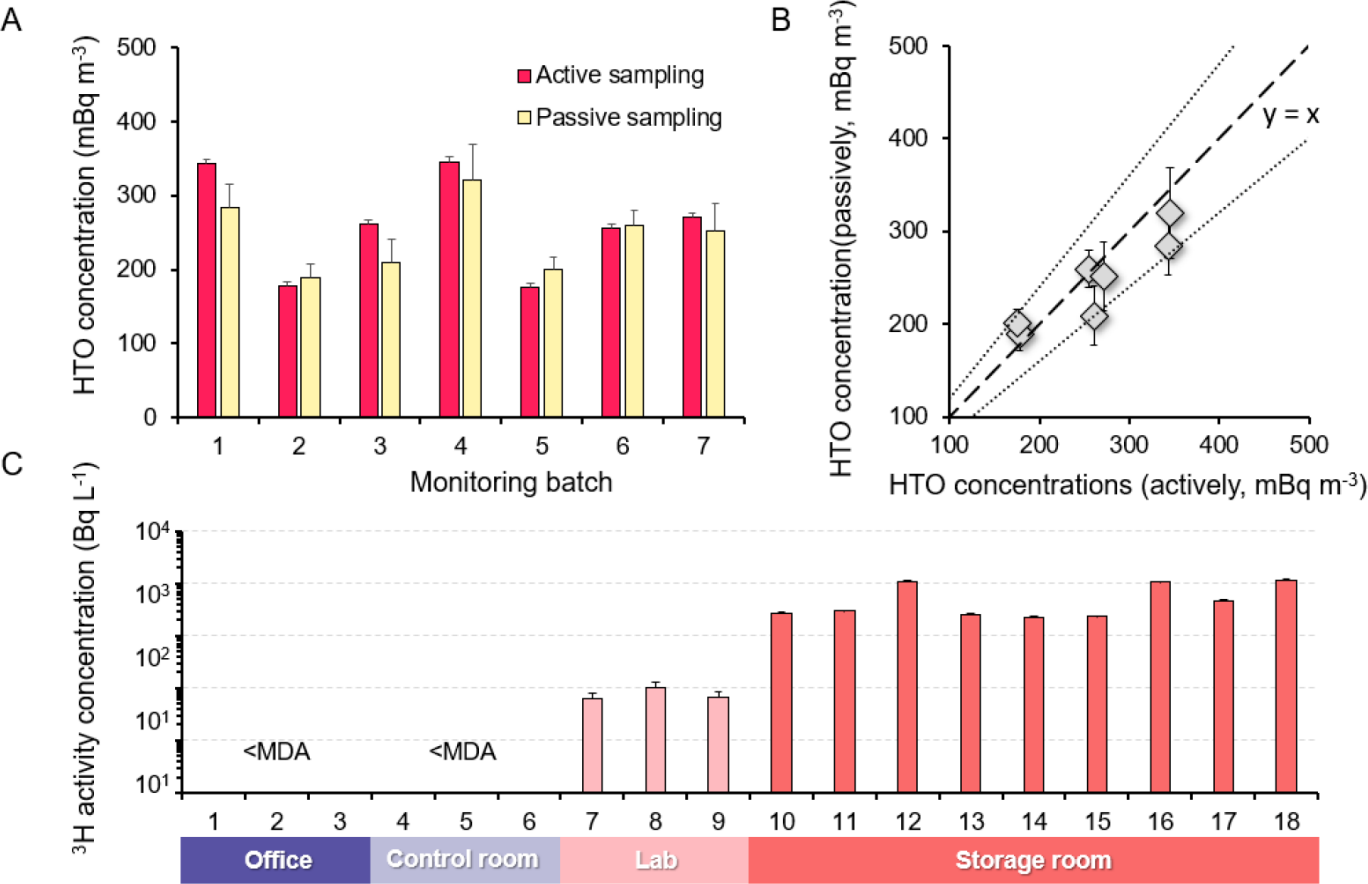
Performance validation of the passive monitoring technique. (A) General comparison of the HTO concentration derived by passive and active sampling simultaneously. The error bars in active and passive groups represent the extended uncertainty (*k* = 2) and standard deviation (*n* = 3), respectively. (B) Linear relationship between the HTO concentration compared between passive sampling and active sampling. Two round-dot lines indicate the boundary of ±20% uncertainty, and the long dashed line is the 1:1 line based on active sampling. (C) Results of the sensitivity validation experiment conducted in indoor environments with varied ^3^H contamination levels. The error bar is the combined uncertainty (*k* = 1) of passive monitoring. The *x*-axis refers to the samples’ IDs.

Influenced by the source term and the exposure environment (e.g., flow field, temperature, humidity), airborne HTO levels often present significant variations over time, which makes it critical to carefully consider the method’s sensitivity when evaluating its practical value, i.e., whether passive monitoring results are sensitive to rapid tritium dynamics. Figure 4A compares the airborne ^3^H concentrations determined by active and passive sampling in 7 days. Overall, the average air concentrations derived from the two methods are consistent, in which the relative standard deviation (*RSD*, in absolute value) is generally within 20% (Figure 4B, 1.59–19.72%, median: 7.1%). A relatively consistent ^3^H concentration among three replicate samples was observed in each batch of the experiment, with a narrow CV range (7.7–15.3%, median: 11%), though the spatial difference in humidity results in a relatively greater variation in gaining weight (0.7–21.3%, median: 6.8%). These results, therefore, demonstrate the feasibility of the passive monitoring technique in independently characterizing ^3^H dynamics in contaminated environments. Further statistical analysis exhibits a significantly negative correlation (*r* = −0.72, *P* < 0.01) between the CV of passive ^3^H data and the RSD in the corresponding monitoring group. This result somehow implies the limitation of the passive monitoring technique; namely, the tritium contents would be slightly underestimated in the scenario with high tritium spatial variability because of its low sampling rate.

While the exposure experiments conducted in different environments also demonstrate the method’s good sensitivity in distinguishing different ^3^H contaminations spatially, the monitoring results (Figure 4C) show that despite taking 24 h of counting, the ^3^H levels in the office and reactor control room are still below the detection limits. These results are generally within our expectations as the continuous ventilation system in the control room and the work area significantly dilutes tritium contamination levels. In contrast, slight ^3^H contaminations (75 ± 22 Bq L^–1^) were determined in the radiochemistry lab, where about 10 GBq of tritium material was used for just a labeling experiment. Even though such contamination is much lower than the control criteria of radioactive contamination or the conservative threshold for safety concerns, our results still add value for proving the great sensitivity of the passive method to screen areas with limited contamination. Furthermore, at a rapid screening condition with 3 h of counting, it is easy to identify relatively high ^3^H contents in the radioactive waste storage room (2930 ± 1209 Bq L^–1^) with an uncertainty below 10%.

### Airborne HTO Dynamics in the Reactor Hall

**Figure 5:**
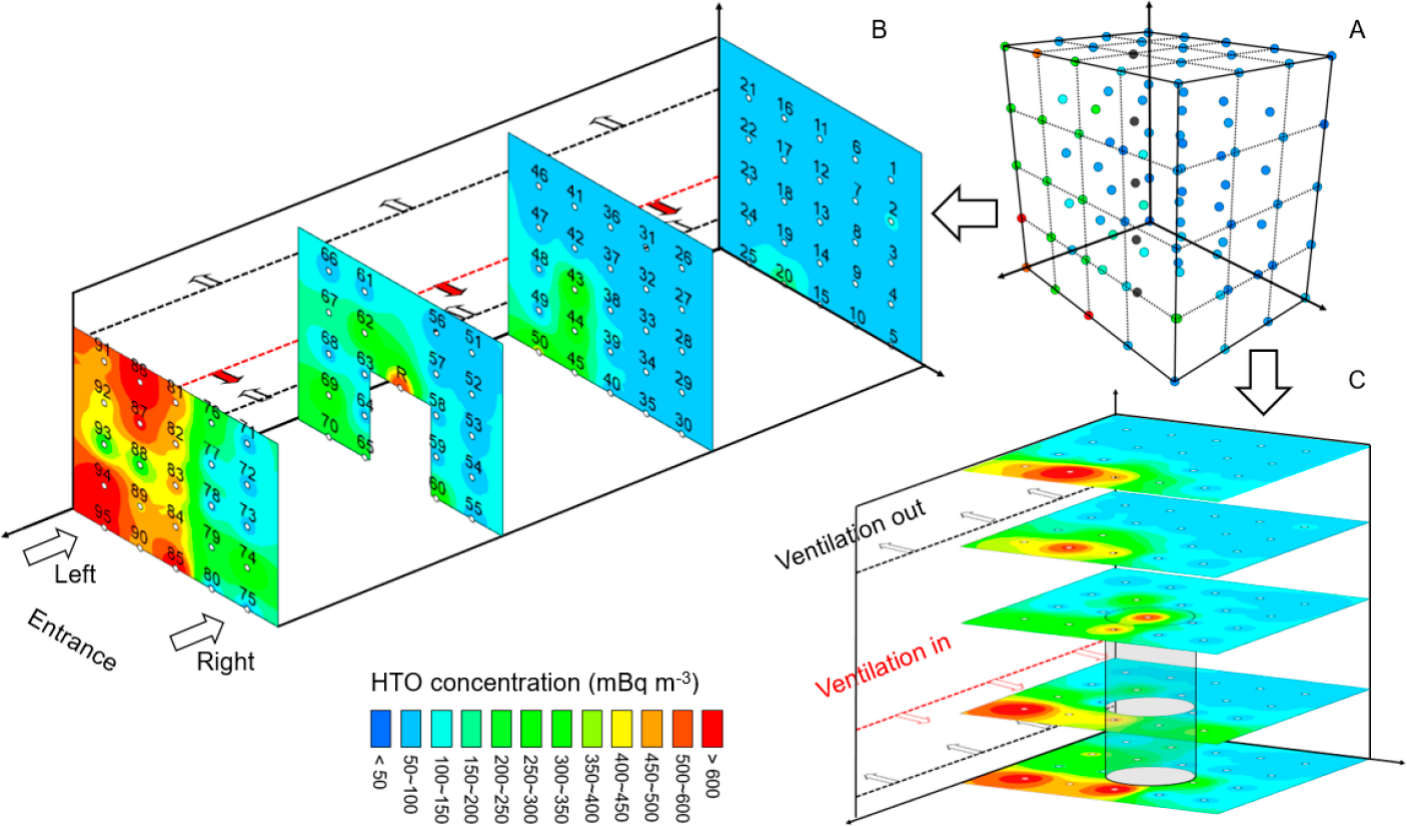
Spatial distribution of airborne HTO concentration in the reactor hall. (A) HTO concentrations at monitoring locations. (B, C) Front and top views of HTO distributions using spatial interpolation. The dotted lines are the ventilation system.

The airborne HTO dynamics at a daily resolution is summarized in Table S5. The collected water vapor in 97/98 of deployed samplers fell within 2.8 g (0.7–3.2 g, median: 1.3 g), indicating the success of our strategy in adjusting the sampler openings to ambient humidity for improving sampling representativeness. In approximately 33% of the prepared samples (32/98), tritium was detectable with a relatively wide variation in HTO specific activity (<MDA – 619 Bq L^–1^, Figure S14). It is unsurprising since HTO could still evaporate into the reactor hall through the pool’s surface even during the shutdown, while the 24-h ventilation system would strongly dilute ^3^H contents in some areas. Nevertheless, given that airborne HTO contents highly depend on the reactor type and their operational status, previous studies have reported significant variability in HTO levels near/within nuclear facilities, for instance, South Korea (24–1513 Bq L^–1^),^53^ France (34–231 Bq L^–1^),^54^ Denmark (20000–40000 Bq L^–1^),^41^ and Japan (300–27000 Bq L^–1^).^55^ Hence, our measured ^3^H contents still fall within the normal range of ^3^H fluctuations. To better understand the tritium spatial distribution in the reactor hall, we set the HTO specific activity and uncertainty at the undetectable points to half of the corresponding MDA.^14^ The HTO volumetric concentrations at all data points (Figure 5A) were then estimated with back-calculated humidity. The spatial distribution profiles of ^3^H were then visualized (front view: Figure 5B and top view: Figure 5C).

Similarly, the HTO volumetric concentrations also exhibit a significant variation, ranging from 75 to 945 mBq m^–3^ (median: 83 mBq m^–3^), which is slightly lower than the values observed in the experimental reactor hall at Kyoto University, Japan (840–3700 mBq m^–3^)^56^ but still higher than the general HTO baseline (around 10 mBq m^–3^).^54^ In the vertical direction, we noted an evident decreasing trend (*P* < 0.01) in HTO of the vertical profile from the entrance (points 71–95, 373 ± 187 mBq m^–3^) to the distal side (points 1–25, 86 ± 11 mBq m^–3^), suggesting the ^3^H accumulation close to the entrance. Furthermore, in this hotspot profile, the average ^3^H concentration on the left side (points 81–95, 547 ± 189 mBq m^–3^) is significantly higher (*P* < 0.01) than the value measured on the right side (points 71–80, 133 ± 93 mBq m^–3^). More interestingly, in the uppermost horizontal monitoring profile, about 8 m above the pool port of the reactor, this decreasing trend in HTO concentration from the entrance to the inside and from left to right still exists. Relatively higher tritium concentration was observed at points of 81 (502 ± 54 mBq m^–3^), 86 (945 ± 55 mBq m^–3^), and 91 (478 ± 54 mBq m^–3^), which are even slightly higher than one point near the reactor pool port (R3, 468 ± 49 mBq m^–3^). A possible explanation for this inhomogeneous spatial distribution is that the different flow rates of the ventilation system result in varied air exchange rates and, in turn, different tritium removal efficiencies at different positions in the reactor hall. In fact, our preliminary investigation on ventilation seemingly supports this hypothesis since the ventilation rates near the entrance are nearly 1 order of magnitude higher than those located on the distal side, and the flow rates on the left side are generally stronger than the right side. Additionally, the profile’s height near the entrance is approximately 2 m lower than the distal one, and the relatively weaker dilution effect by the air column may further increase the potential for regional tritium hotspots. Without overemphasizing the conclusion, our work suggests the possibility of inhomogeneous pollution distribution in the large hall and hence secondary contamination hotspots due to the flow field difference. Further efforts, such as field investigation and modeling, are thus needed to study the transport process of HTO evaporated from the reactor pool in the hall.

### HTO Release Rate and Occupational Exposure Risk

The airborne HTO inventory in the reactor hall was estimated to be approximately 1.23 ± 0.06 MBq, with an average volumetric HTO concentration of approximately 172 ± 9 mBq m^–3^. Using the mass balance model, we estimated the total ^3^H release rate of the reactor hall in the steady-state case to be approximately 3199 ± 306 Bq h^–1^. To assess whether additional tritium release exists in the reactor hall, we further estimated the HTO evaporation rate from the reactor pool port, which is thought to be the dominant contributor during reactor shutdown. The investigation shows that the HTO specific activity in the pool’s surface water was about 2078 ± 20 Bq L^–1^, and the tritium input through the pool’s surface evaporation of about 2947 ± 254 Bq h^–1^ could be thus obtained by using the simultaneous temperature and humidity monitoring data. The relative deviation between the theoretical and measured values of the total ^3^H source intensity in the reactor hall is about 10%, which may be caused by an overestimation of ^3^H concentration in the measured data as we set the undetectable data to half of MDA. Hence, our result indicates the dominant contribution of tritium input by pool evaporation and demonstrates the TRIGA II reactor’s robust operational status.

The investigation conducted on the ground-floor work area shows no significant difference (*P* = 0.21) in HTO concentration (Figure S15) during operation (254 ± 99 mBq m^–3^, *n* = 20) and shutdown (228 ± 156 mBq m^–3^, *n* = 20), which further supports the above inferences. It is well-known that tritium is produced by neutron activation in the primary coolant (i.e., pool water) in the TRIGA II reactor, and the anthropogenic tritium mainly presents in the form of tritium gas (i.e., HT or T_2_) in initial rather than tritiated water. Our previous work observed and analyzed tiny bubbles in the surface pool during reactor operation.^57^ The similar tritium levels at the two stages could thus be attributed to the rapid removal of newly produced tritium gas by the ventilation system before its oxidation. From the viewpoint of radiation protection, the internal exposure risks by ^3^H at two stages (Figure S16) are limited as the maximum dose contribution (1.4 μSv y^–1^) remains about 4 orders of magnitude lower than the occupational dose limit (20 mSv y^–1^). Nevertheless, it is interesting that HTO levels tend to be relatively higher at the southwest corner (i.e., left entrance) during operation and shutdown, suggesting that the flow field played a more critical role in HTO distribution than tritium input. Therefore, deploying online tritium monitoring instruments at the tritium hotspot area rather than a random location would improve the efficiency of nuclear safety monitoring. It is also noteworthy to take more care of these tritium hotspots in future nuclear decommissioning owing to the more severe radiation exposure that the materials may get.

## Implications

Recently, the discharge of radioactive wastewater from Fukushima into the Pacific Ocean has triggered major concerns about tritium and other radionuclides to the public and the scientific communities.^58−60^ In the near future, it is expected that anthropogenic ^3^H release will keep increasing. For example, some countries are now reconsidering the use of nuclear power against climate change and the energy crisis,^61^ with 57 nuclear reactors being under construction worldwide.^62^ Moreover, the first net energy gain of fusion reaction implies significant progress in nuclear fusion technology,^63^ which might cause additional tritium input into the environment.^11^ Therefore, enhanced control measures of environmental tritium, especially inside nuclear facilities, are an intrinsic part of the responsible utilization of nuclear energy.

Based on a new passive sampler design and a tailored HTO analytical protocol, this study presents a reliable, efficient, and representative passive monitoring technique, enabling the quantification of airborne tritium contamination in any indoor environment with high spatial and temporal resolution. Unlike the conventional localization of tritium contamination on a 2D profile, we provided the 3D spatial distribution of HTO in a nuclear reactor hall for the first time and reveal the tremendous spatial heterogeneity of HTO. In addition, we also contribute vivid lessons on the application of passive monitoring schemes in estimating the tritium release rate and the resulting occupational exposure risk. It not only provides a low-cost and convenient tool to regulators to better diagnose the operational status of nuclear facilities based on the recorded HTO fluctuations but also offers more and reliable information to policymakers to establish targeted control strategies to address future tritium contamination challenges. Moreover, it is worth underscoring that the 3D monitoring matrix demonstrated here and its applications will also provide new insights into monitoring and controlling other indoor pollutants.

Although the internal exposure risk by tritium is negligible in our case, radiation protection concerns for HTO should still be aware in some environments with heavy water reactors or some compact nuclear facilities, in which HTO levels were reported to be approximately 7 orders of magnitude higher than the HTO baseline.^64^ Apart from this, another significance of this work is that we improve the visibility of environmental tritium contamination from 2D to 3D, thus opening a new door for taming radioactivity. We believe that the best way to counter any unjustified fears of radiation is by providing comprehensive information about its level and risk based on scientific knowledge. Hence, the passive monitoring technique may contribute to the construction of a practical culture of radiation protection, which is essential for efficient communication with the public as well as for policy implementation and enforcement.^65^

## Supporting Information Available

Batch experiments for optimizing leaching condition (part 1); ^3^H analysis and uncertainty assessment (part 2); Validation of radiometric properties of leaching method (part 3); Evaluation of passive monitoring technique (part 4); Reactor investigation and model calculation (part 5); Exposure risk estimation (part 6); QA/QC (part 7); Characteristics of leachate under different conditions (part 8); Flowchart of the optimized leaching method (part 9); Results of radiometric properties of the leaching method (part 10); Results of reactor investigation (part 11); and Spatial profiles of HTO and radiation exposure in the ground-floor work area (part 12) (PDF)
