# Supplementary material for: Development of a Novel Passive Monitoring Technique to Showcase the 3D Distribution of Tritiated Water (HTO) Vapor in Indoor Air of a Nuclear Facility
Source: Environ Sci Technol. 2023 Nov 15;57(48):20024–33. doi: 10.1021/acs.est.3c05783 (PMC10702432; doi:10.1021/acs.est.3c05783)
Supplement: Supplementary file 1 — es3c05783_si_001.pdf [file es3c05783_si_001.pdf]

Development of a Novel Passive Monitoring Technique to Showcase the 3D Distribution of Tritiated Water (HTO) Vapor in Indoor Air of a Nuclear Facility

Bin Feng[1,2], Martin Ibesich[3], Dieter Hainz[3], Daniel Waidhofer[3], Monika Veit-Öller[3], Clemens Trunner[3], Thomas Stummer[3], Michaela Foster[3], Markus Nemetz[3], Jan M. Welch[3], Mario Villa[3], Johannes H. Sterba[3], Andreas Musilek[3], Franz Renz[2], Georg Steinhauser[1]*

1. TU Wien, Institute of Applied Synthetic Chemistry & TRIGA Center Atominstitut, TU Wien, 1060 Vienna, Austria
2. Institute of Inorganic Chemistry, Leibniz Universität Hannover, 30167 Hannover, Germany
3. TRIGA Center Atominstitut, TU Wien, 1020 Vienna, Austria

Corresponding author: georg.steinhauser@tuwien.ac.at

This Supporting Information consists of 12 parts, including 7 descriptive texts, 5 tables, and 16 figures.

Supporting Information Guide

1. M&M: Batch experiments for optimizing leaching condition

- **Text 1.** Descriptions regarding batch experiments with different leaching conditions.

2. M&M: ^3H analysis and uncertainty assessment

- **Text 2**. Descriptions regarding tritium analysis procedure and uncertainty assessment of measured data

3. M&M: Validation of radiometric properties of leaching method

- **Text 3.** Descriptions regarding validation experiments of radiometric properties (linearity, reproducibility, and MDA) of leaching method.
- **Figure S1.** Photo of the active and passive monitoring instruments used in co-comparison experiment.

4. M&M: Evaluation of passive monitoring technique

- **Text 4.** Descriptions regarding experiments (co-comparison experiments with active monitoring and sensitivity test) of passive monitoring technique.
- **Figure S2.** Exposure experiments conducted in different rooms at the TRIGA Center Atominstitut, TU Wien

5. M&M: Reactor investigation and model calculation

- **Text 5.** Descriptions regarding the analysis of data obtained from reactor investigation and the estimation of HTO release rate by the mass balance model and theoretical evaporation calculation.
- **Figure S3.** 3D monitoring matrix for airborne HTO investigation in the reactor hall of TU Wien.

- **Figure S4.** Explanation for the airborne HTO inventory estimation in the reactor hall.
- **Table S1.** The parameters and uncertainty used in theoretically estimating HTO evaporation rate.

6. M&M: Exposure risk estimation

- **Text 6.** Descriptions regarding the estimation of occupational exposure risks induced by HTO during operation and shutdown.

7. M&M: QA/QC

- **Text 7**. Descriptions of QA/QC measures adapted in this study.
- **Table S2.** Equilibrium load of zeolite 4Å used in different batches of experiment.
- **Table S3.** Background counting rate for Tri-Carb 2910RT and Hidex 300 SL in our laboratory.
- **Figure S5.** Cross comparison of ^3H measurement in different LSC systems using AGES sample.
- **Figure S6.** Cross comparison of ^3H measurement in different LSC systems using ATI sample.

8. R&D: Characteristics of leachate from different adsorbents

- **Figure S7**. Apparent effects of leaching amount on different adsorbents and swelling effect after adding triple distilled water in silica gel without color indicator.
- **Figure S8**. The transparency of leachate before and after using filter.
- **Table S4**. Extracted water and conductivity in different leaching amounts.

9. R&D: Flow chart of the optimized leaching method

- **Figure S9.** Flow chart of the optimized leaching method.

10.R&D: Radiometric properties of leaching method

- **Figure S10.** The linear relationship between the ^3H specific activity in the leachate and the spiked ^3H of the standard.

- **Figure S11.** The linear relationship between the ^{3}H specific activity in the leachate and the spiked ^{3}H of the standard after one month and four months of preparation.
- **Figure S12.** Recovery of ^{3}H under different leaching durations.
- **Figure S13.** The method's MDA in Hidex 300SL and Tri-Carb 2910RT.

11.R&D: Reactor investigation

- **Table S5.** Detailed information on airborne HTO in the reactor hall.
- **Figure S14.** Spatial distribution of HTO specific activity in different vertical profiles.

12.R&D: Spatial profiles of HTO and dose rate in the ground-floor work area

- **Figure S15.** Spatial distribution of airborne HTO concentration in the ground-floor work area at operation and shutdown stages.
- **Figure S16.** Spatial distribution of annual accumulated dose in the ground-floor work area at operation and shutdown stages.

1. M&M: Batch experiments for optimizing leaching condition

In recent decades, several analytical protocols have been developed to measure ^{3}H contents in radioactive solid waste materials to meet the requirements of ^{3}H analysis in nuclear decommissioning.[1] For example, Kim et al. [2] proposed the analytical method for accurate ^{3}H determination in the bio shield concrete; Croudace et al. [3] proposed a protocol for ^{3}H speciation analysis in the contaminated metals. When decommissioning heavy water nuclear reactors, Das and Hou utilized the silica gel as a medium to quantify the ^{3}H levels in the contaminated environment[4]. Due to the high tritium contamination levels encountered in these sites, diluting the samples before performing LSC counting is often necessary. Hou et al. suggested a leachate-to-sample ratio to be 3 for the silica gel obtained from the heavy water reactors.[1] However, using existing protocols, the lower tritium content in air and limited water samples collected through the passive sampler present challenges to accurately determining the tritium content in lightly contaminated samples, as a higher leaching ratio would result in a stronger ^{3}H dilution effect. Hence, developing a new leaching protocol targeting passively collected samples would be more conducive to convenient sample preparation and sufficient detection sensitivity.

In this study, we systematically investigated leachate characteristics under various leaching conditions, including leaching materials, amount, duration, and temperature. Based on the experimental results, we designed and optimized a new leaching protocol for better preparing passively collected samples. Three replicate samples were employed in each batch of experiments to assess the sample variability. The leachate recovery and the quenching level in the leachate were considered the quantitative indices of the method's operability and reproducibility. Ideally, we hope to obtain a sufficient volume of leachate (i.e., approximately 10 mL) with a low quenching effect for further reliable ^{3}H analysis.

First, we investigated the difference in the leachate properties among three common adsorbents, including zeolite 4 Å (Disidry® Silicagel, 3 – 5 mm), silica gel without a color indicator (Supelco®, 2 – 5 mm), and orange silica gel with a cobalt indicator (Carl Roth GmbH, 2 – 5 mm). Approximately 30 g of adsorbent was placed in a sealable beaker, where about 35 g of triple distilled water was added in advance. After that, the beaker was immediately sealed, and a sealing film (PARAFILM®) was used to prevent air exchange. After one day of leaching, all residual leachate was extracted with a syringe, and the collected volume was weighed with a balance (resolution: 0.01 g). The leachate was then filtered by a syringe filter and the LSC sample was prepared using about 10 mL of filtered leachate mixed with 10 mL of scintillation cocktail (Ultima Gold LLT). The quenching index, tSIE, in Tri-Carb 2910RT was used to quantify the quenching level of each prepared sample. Based on the qualitative observation of the leachate and two quantitative indices (details see Figure 3a), we demonstrated that the zeolite was more appropriate in our leaching experiment.

The performance of zeolite leachate after adding different amounts of leaching water was subsequently investigated. Similarly, about 30 g of zeolite was added to the sealable beaker, where a given amount of triple distilled water (10, 20, 25, 27, 30, 35, 40, 45, and 50 mL) was set up. After one day, the leachate was collected, and its volume was weighed. Moreover, a calibrated conductivity meter (Xylem Analytics Germany) was used to quantify the impurity levels in the leachate before and after filtration. About 10 mL of filtered leachate was mixed with the cocktail for later LSC counting.

A two-week leaching experiment was carried out to learn how leaching duration affects the characteristics of the leachate. Approximately 30 g of zeolite was packed in a sealable beaker with 35 mL of triple distilled water, and the leachate was then collected at 1, 2, 3, 4, 5, 7, and 14 days

after the start of the experiment. The collected volume, conductivity, and quenching index of leachate were quantified as described above. In addition, the diluted tritium standard water (approximately 79.1 ± 1.2 Bq L^{-1} as of December 2022) was prepared to verify the recovery efficiency of tritium. About 35 mL of triple distilled water was added into a sealed beaker that about 2 mL of tritium standard water and 30 g of zeolite had reached an equilibrium. Following a similar experimental procedure, the leachate with different leaching duration was collected, and the ^3H levels in the leachate were determined using the LSC systems for 12 h.

Furthermore, the influence of leaching temperature on the leachate's quenching level was also investigated. The sealable beaker loaded with 30 g of zeolite and 35 mL of triple distilled water was placed in a water bath with constant temperature (50°C and 80°C) for one day and four days of leaching. The opening of the bath was covered with aluminum foil to prevent water evaporation. Following the above procedure, the LSC sample was prepared using the filtered leachate, and the corresponding tSIE was determined by Tri-Carb 2910RT.

REFERENCE:

(1) Hou, X. Radiochemical Analysis of Radionuclides Difficult to Measure for Waste Characterization in Decommissioning of Nuclear Facilities. *J. Radioanal. Nucl. Chem.* **2007**, *273*, 43–48. https://doi.org/10.1007/s10967-007-0708-x.

(2) Kim, D.; Croudace, I. W.; Warwick, P. E. The Requirement for Proper Storage of Nuclear and Related Decommissioning Samples to Safeguard Accuracy of Tritium Data. *J. Hazard. Mater.* **2012**, *213–214*, 292–298. https://doi.org/10.1016/j.jhazmat.2012.01.094.

(3) Croudace, I. W.; Warwick, P. E.; Kim, D. Using Thermal Evolution Profiles to Infer Tritium Speciation in Nuclear Site Metals: An Aid to Decommissioning. *Anal. Chem.* **2014**, *86*, 9177–9185. https://doi.org/10.1021/ac502244a.

(4) Das, H. A.; Hou, X. Steady-State Leaching of Tritiated Water from Silica Gel. *J. Radioanal. Nucl. Chem.* **2009**, *280*, 467–468. https://doi.org/10.1007/s10967-009-7458-x.

2. M&M: ^{3}H analysis and uncertainty assessment

In our lab, a mixing ratio of 10:10 with the cocktail (Ultima Gold LLT, PerkinElmer) in a 22 mL polytetrafluorethylene (PTFE) counting vial was used for the sample preparation. Two calibrated LSC systems, Tri-Carb 2910RT (PerkinElmer) and Hidex 300SL (Hidex) was employed in the ^{3}H analysis. The counting window for the LSC system was optimized to achieve low MDA. Before counting, a 24-hour dark adaption was performed on all the prepared samples to reduce the potential influences caused by chemiluminescence and photoluminescence. Usually, the total counting time was divided into ten sub-counting durations with the same counting interval. LSC quenching curve calibration used the ^{3}H reference solutions (IAEA) and quenching agent (CCl_4). The leachates produced by triple distilled water were employed as the blank sample. Therefore, the ^{3}H activity concentration in the investigated indoor environment (A in Bq L^{-1}) can be estimated by the Equation (1):

$$A = \left(\frac{C_\mathrm{s} - C_\mathrm{b}}{\varepsilon \times 60 \times m_\mathrm{s}}\right) \times \frac{(m_\mathrm{a} + m_\mathrm{l})}{m_\mathrm{a}} \quad (1)$$

where C_s and C_b are the count rate (cpm) of environmental sample and blank; ε represents the counting efficiency that is given by calibrated quenching curve; m_s, m_a, and m_l are the volumes of LSC sample (mL), passively collected sample (mL) and leaching amount (mL).

Referring to our previous work,[1] the combined uncertainty in ^{3}H activity (U, $k = 1$) due to the errors caused by the components in Equation 1 was estimated by the error propagation equation (Equation 2). For the radiation measurement, the error due to statistical fluctuations during counting time was estimated on the Poisson distribution (Equation 3). With respect to the introduced error during sample weighing, the standard deviation of multiple measurements was

used as the error. The 95% confidential interval of the calibrated quenching curve was adopted to estimate the error of counting efficiency at different quenching levels.

$$U = \sqrt{\sum\left(\frac{\partial A}{\partial x_i} \times u(x_\mathrm{i})\right)^2} \quad (2)$$

where $\partial A/\partial x_\mathrm{i}$ and u (x_i) are the sensitive coefficient and the standard uncertainty for each error component, respectively.

$$U(C) = \sqrt{\frac{C_\mathrm{i}}{t_\mathrm{i}}} \quad (3)$$

where C_i and t_i are the counting rate (cpm) and counting time (min) of the prepared LSC samples (i.e., environmental samples and blank samples).

REFERENCE

(1) Feng, B.; Chen, B.; Zhao, C.; He, L.; Tang, F.; Zhuo, W. Application of a Liquid Scintillation System with 100-ml Counting Vials for Environmental Tritium Determination: Procedure Optimization, Performance Test, and Uncertainty Analysis. *J. Environ. Radioact.* **2020**, *225*, 106427. https://doi.org/10.1016/j.jenvrad.2020.106427.

3. M&M: Validation of radiometric properties of leaching method

To test the linearity of the leaching method, diluted ^{3}H standards with varied activities (~80, 100, 500, 5000, and 10000 Bq L^{-1}) were prepared using mother ^{3}H standard and triple distilled tap water. In each ^{3}H concentration level, about 2 mL of the ^{3}H standard was injected into a beaker, and adding amount was weighted by the balance (Sartorius GmbH, 0.01 mg). Subsequently, about 30 g of zeolite 4 Å was loaded, and the beaker was then sealed for 24 h. The established leaching protocol was applied for sample preparation and ^{3}H analysis. The isotopic effect was quantified by the slope (i.e., η) of linear regression ($Y = \eta \cdot X$) between the ^{3}H activities in the leachate (X in Bq L^{-1}) and the added solution (Y in Bq L^{-1}). Three replicate samples were prepared to evaluate reproducibility. In addition, these samples were remeasured at one and four months after preparation for assessing their long-term stability. The minimum detectable activity (*MDA*) of the leaching method with counting time (t in min) was estimated by Equation (4):

$$MDA = \frac{\left(2.71+4.65\sqrt{C_{\mathrm{b}}\times t}\right)}{\varepsilon\times 60\times m_{\mathrm{s}}\times t}\times 1000\times\frac{(m_{\mathrm{a}}+m_{\mathrm{l}})}{m_{\mathrm{a}}} \tag{4}$$

where C_{b} are the count rate (cpm) of the blank sample; ε represents the counting efficiency (%); m_{s}, m_{a}, and m_{l} are the volumes of the LSC sample (mL), passively collected sample (mL), and leaching amount (mL).

4. M&M: Evaluation of passive monitoring technique

Before the application in the reactor hall, the feasibility of the developed technique was validated by an active sampling system consisting of a well-calibrated sampling pump (AirChek TOUCH, SKC) and three-stage gas-wash bottles. A 7-day comparison experiment was conducted in the ^{3}H-contaminated environment where three passive samplers and the active sampling system were simultaneously deployed for collecting the airborne HTO daily.

Figure S1 shows a homemade active sampling system, and three replicate passive samplers (diameter of 40 mm) were prepared in each experiment batch. In the active sampling system, about 300 mL of triple distilled water was assigned into three gas wash bottles to collect atmospheric HTO vapor (efficiency over 99%).[1] A stable flow rate of 2 L min^{-1} was adopted in all batches of active monitoring. The used bottles were cleaned three times with distilled water after the monitoring campaign to reduce the memory effect of tritium from the previous sampling batches. In the passive sampling system, about 30 g of zeolite 4 Å (spherical 3 – 5 mm) was equipped in the passive sampler before use, and the QA/QC measures in sample transportation and storage were also considered in this section.

After sampling, the accumulated sampling volume shown on the pump was recorded, and the residual water in three bottles was gathered together in a new container. Approximately 10 mL of water was transferred into a 22 mL counting vial and then mixed with a 10 mL scintillation cocktail (Ultima Gold LLT). With the total water volume and accumulated sampling volume, the HTO concentration in the air could then be estimated. For the passive samples, the established protocol was used for sample preparation and analysis. The ^{3}H activities in these prepared samples were measured by the Tri-Carb 2910RT for 7 – 12 h. In each counting batch, a blank sample was accompanied by measurement for a dynamic background counting rate.

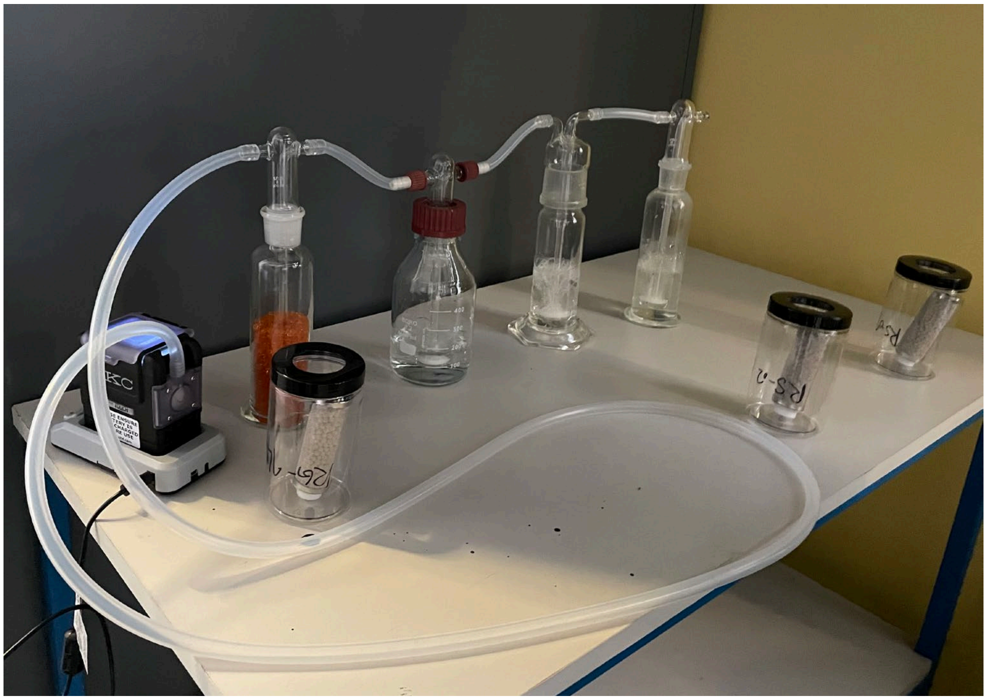

Figure S1. The active and passive monitoring instruments used in the co-comparison experiment.

Besides, the technique's sensitivity was also examined in this study. The passive samplers were deployed in different rooms (Figure S2) at TRIGA Center Atominstitut, TU Wien, to test the distinguishing ability of the passive monitoring technique for tritium contaminations. Following the radiation protection regulation, the four rooms under investigation equip a 24-hour ventilation system. However, due to the presence of potential ^3H sources in some rooms, a significant difference in their ^3H levels is expected.

In the office (Figure S2A) and the reactor control room (Figure S2B), we considered the tritium levels to be relatively low as there are no tritium sources in the surrounding area. While in the radiochemical laboratory (Figure S2C), we expected there would be some risks of ^3H contamination since a tritium labeling experiment was just conducted in the room a few days before our investigation. In contrast, the radioactive waste storage room (Figure S2D) was

considered as relatively heavily tritium contaminated because of some contaminated materials and a sealed tritium source container in the room.

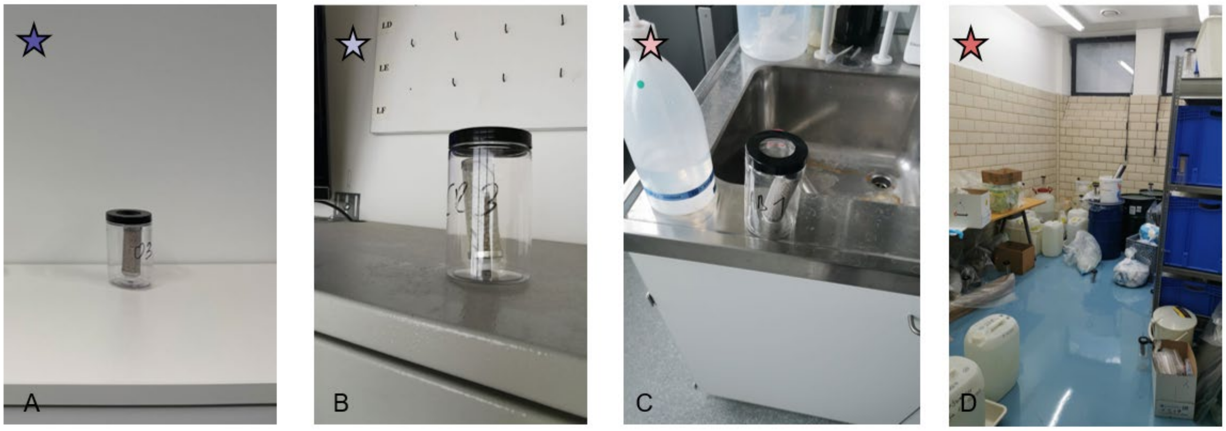

Figure S2. Exposure experiments conducted in different rooms at the TRIGA Center Atominstitut, TU Wien. (A) an office; (B) reactor control room; (C) radiochemical laboratory; (D) radioactive waste storage room.

REFERENCE

(1) Munyon, W. J.; Reilly, D. W.; Webb, J. AGHCF Gaseous-Effluent Tritium Sampling System: Design Considerations and Performance Testing Results. *Health Phys.* **2008**, *94*, 75–85. https://doi.org/10.1097/01.HP.0000279630.18757.80.

5. M&M: Reactor investigation and model calculation

Based on the passive monitoring matrix (Figure S3), we have divided the reactor hall into about 95 sub-volumes. The midpoint connection between each sampler and the adjacent sampler is considered the virtual boundary, so each sub-volume could be estimated using the measured dimensional data (Figure S4). It should be noted that the height difference of about 2 m in the roof from the entrance to the distal end was considered in the calculation, resulting in the larger volume of those "boxes" located at the topmost layer than those in the lower layers. Limited by the spatial resolution of the monitoring matrix, the airborne HTO concentration (mBq m^{-3}) was assumed homogeneous within each sub-volume. Therefore, the total HTO inventory in the air (I, Bq) could be estimated by Equation 4. Using Equation 5, the volume-weighted airborne HTO concentration (C_v, mBq m^{-3}) can be calculated by the HTO inventory and the total volume of the reactor hall (V, m^3).

$$I = \sum_{i=1}^{95}(L_i \times W_i \times H_i \times C_i) \quad (4)$$

$$C_v = \frac{I}{V} \quad (5)$$

where L_i, W_i, and H_i are the length (m), width (m), and height (m) of a specific sub-volume box. C_i is the corresponding volumetric HTO concentration (mBq m^{-3}). The uncertainty caused by the tritium measurement was considered in the error propagation equation (equation 2) for the total uncertainty estimation in the HTO inventory and volume-weighted HTO concentration (k =1).

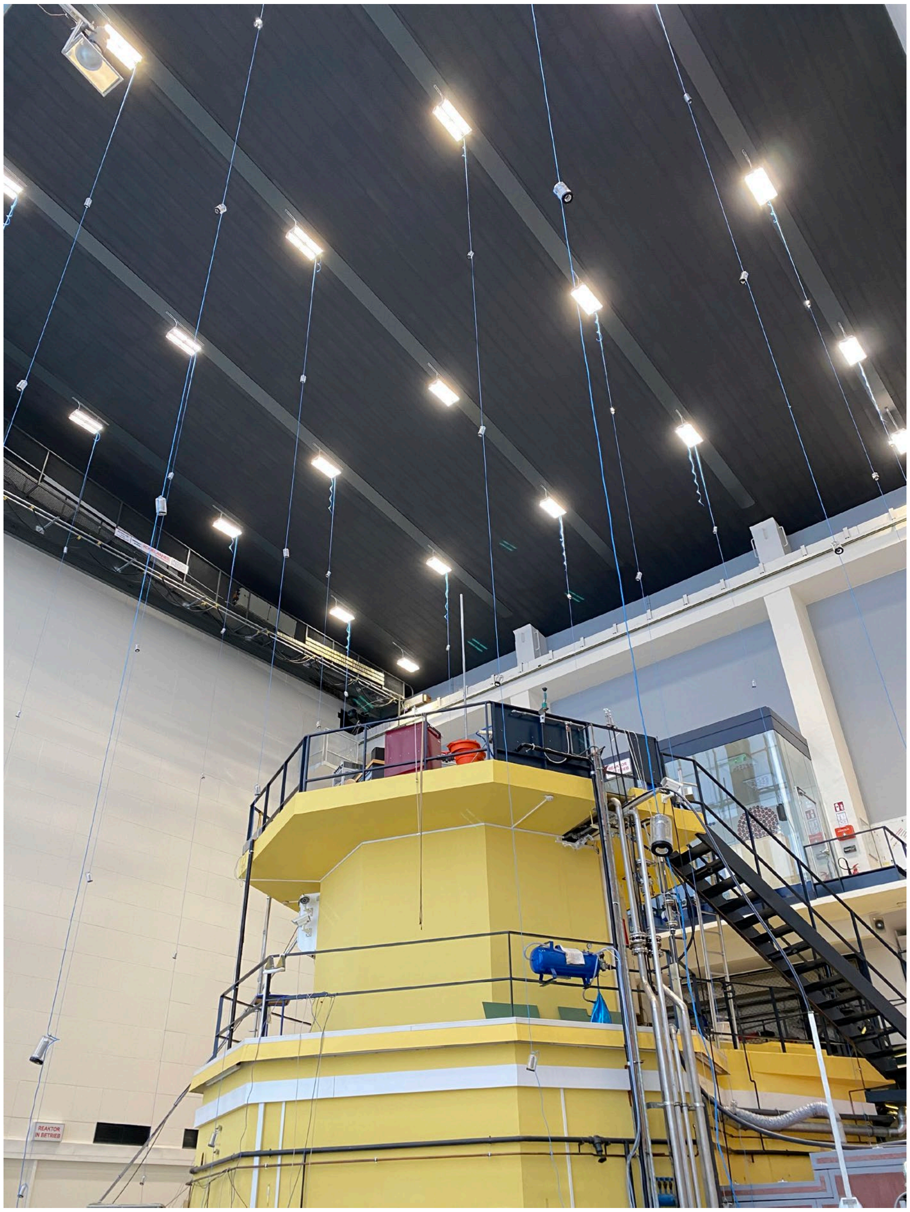

Figure S3. 3D monitoring matrix for airborne HTO investigation in the reactor hall of TU Wien.

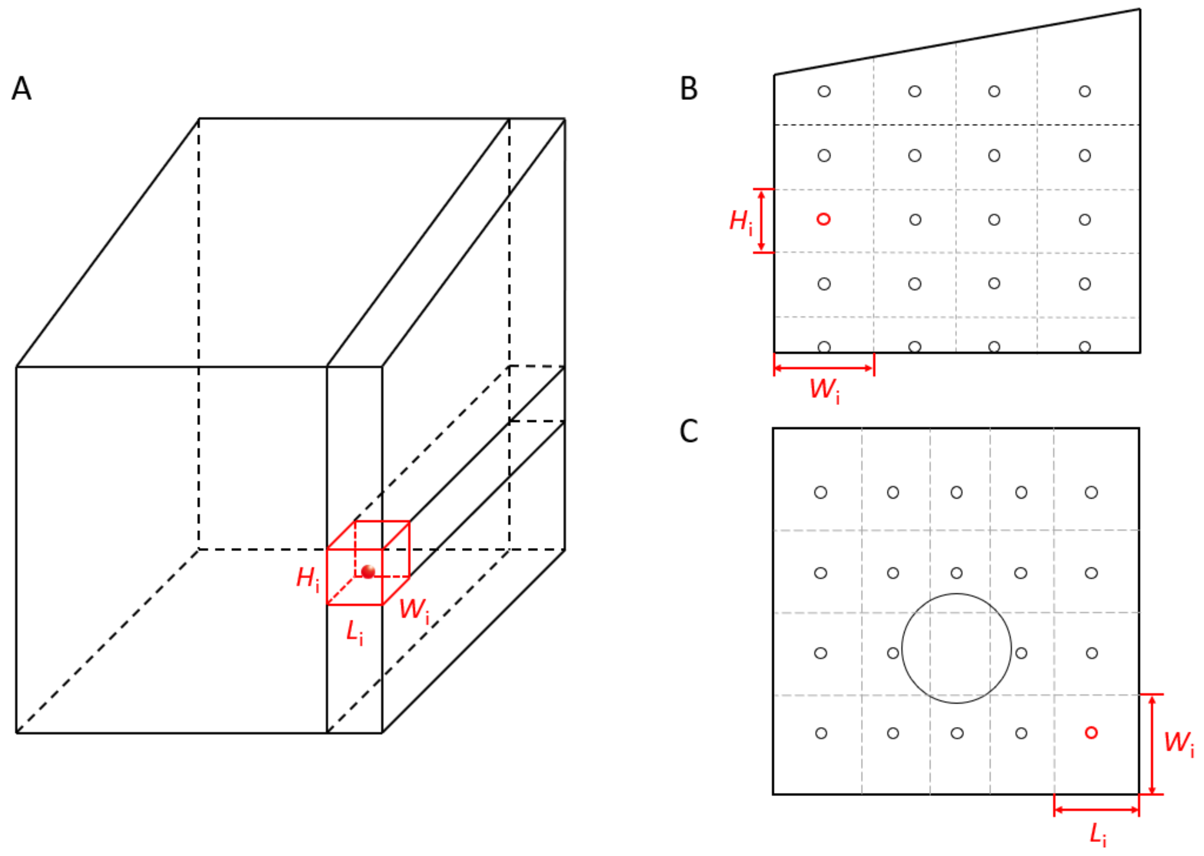

Figure S4. Explanation for the airborne HTO inventory estimation in the reactor hall. (A) Schematic diagram of a sub sampling volume. (B) and (C) are the side view and top view of the monitoring profile. The red point represents the location of a passive sampler.

To estimate the HTO release rate in the reactor hall, the one-compartment mass balance model was performed in this study which has been widely used for estimating the pollutant flux in the indoor environment. In our scenario, because there is no tritium generation during the shutdown stage and dominant tritium input was contributed by the evaporation, we consider the tritium emission was at steady-state. Therefore, according to the description by Blondel and Plaisance,[1] the volume-weighted airborne HTO concentration for the steady-state case can be obtained from the following equation:

$$C_{\mathrm{v}} = \frac{aP}{a+k} C_{\mathrm{o}} + \frac{Q_{\mathrm{HTO}}}{V} \times \frac{1}{a+k} \tag{6}$$

where a is the air exchange rate by the ventilation system (h^{-1}), and k is the net rate of the removal process by physical decay of tritium (h^{-1}). P is the fraction of outdoor ^{3}H penetrating the room (100%), and C_o is the ^{3}H concentration in the outdoor environment (~ 6 mBq m^{-3}) of ATI. Q_{HTO} is the total HTO release rate in the reactor hall (Bq h^{-1}).

Considering that the ventilation rate in the reactor hall (2.77 h^{-1}) is about five orders of magnitude higher than the ^{3}H removal process by physical decay (6.42×10^{-6} h^{-1}), Equation 6 was then simplified. Therefore, the total HTO release rate can be calculated by Equation 7, and the total uncertainty of Q (k = 1) was estimated using the error propagation equation with the error from the available components.

$$Q = (C_V - PC_o) \times V \times a \quad (7)$$

To verify whether or not there is a significant tritium leak from the nuclear reactor, we estimated the tritium release rate by the theoretical evaporation model, in which the HTO evaporation by the reactor pool is hypothesized to be the dominant ^{3}H input in the reactor hall. Because the tritium has similar physicochemical properties as hydrogen, we assume the evaporation of HTO is similar to the water evaporation. In this case, by using the American Society of Heating and Air-Conditioning Engineers (ASHRAE) handbook method (Equation 8),[2] the evaporation rate of water vapor ($Q_{water\ vapor}$, kg h^{-1}) from the opening pool with a diameter of 2 m can be obtained.

$$Q_{water\ vapor} = 0.000144 \times (P_{pool} - P_{air}) \times \left(\frac{2}{2} \times \pi\right) \quad (8)$$

where 0.000144 is the recommended coefficient in the low air velocity range (0.05 – 0.15 m s^{-1}); P_{pool} is the saturated pressure of water vapor (in Pa) in the reactor pool; The P_{air} is the partial pressure of water vapor in the air near the reactor pool (in Pa); ($2/2*\pi$) is the opening area (in m^2) of the pool. With the concurrently recorded environmental parameters (e.g., temperature and

relative humidity) inside/near the reactor pool, the Magnus-Tetens approximation (Equation 9)[3] was performed here to estimate the saturated pressure of water vapor (P_s, Pa) under a specific temperature (T, °C).

$$P_s = 0.61078 \times e^{\left(\frac{17.27 \times T}{(T + 237.3)}\right)} \times 1000 \quad (9)$$

With the measured ^3H specific activity in the reactor pool (A_p, Bq L^{-1}) during our investigation, the theoretical HTO release rate by the water evaporation ($Q_{HTO\text{-}t}$, Bq h^{-1}) can be estimated following by Marang et al. (Equation 10).[4] Note, the specific activity of ^3H in the reactor pool is considered spatially homogenous because of the operation of the internal circulation pump. Table S1 summarizes the parameters and their uncertainty used in the model calculation, and the error propagation equation was used in the total uncertainty estimation (k =1).

$$Q_{HTO-t} = Q_{water\ vapor} \times A_p \quad (10)$$

Table S1. The parameters and uncertainty used in theoretically estimating ^3H evaporation rate.

No.	Parameter	Parameter value	Uncertainty
1	Pool water temperature	29 °C	1 °C
2	Air temperature near the pool	23 °C	1 °C
3	Air relative humidity	31 %	1 %
4	^3H specific activity in the reactor pool	2078 Bq L^{-1}	20 Bq L^{-1}

REFERENCE

(1) Blondel, A.; Plaisance, H. Screening of Formaldehyde Indoor Sources and Quantification of Their Emission Using a Passive Sampler. *Build. Environ.* **2011**, *46*, 1284–1291. https://doi.org/10.1016/j.buildenv.2010.12.011.

(2) Shah, M. M. Improved Method for Calculating Evaporation from Indoor Water Pools. *Energy Build.* **2012**, *49*, 306–309. https://doi.org/10.1016/j.enbuild.2012.02.026.

(3) Murray, F. W. On the Computation of Saturation Vapor Pressure. *J. Appl. Meteorol.* **1967**, *6*, 203–204.

(4) Marang, L.; Siclet, F.; Luck, M.; Maro, D.; Tenailleau, L.; Jean-Baptiste, P.; Fourré, E.; Fontugne, M. Modelling Tritium Flux from Water to Atmosphere: Application to the Loire River. *J. Environ. Radioact.* **2011**, *102*, 244–251. https://doi.org/10.1016/j.jenvrad.2010.11.015.

6. M&M: Exposure risk estimation

In the investigated reactor hall, the ground floor is set up as a work area for the physics group; sometimes, the reactor group employees also work there. In order to quantify the internal exposure caused by ^{3}H release from the reactor to these occupational employees, a survey targeted at the groundwork area was conducted during the operation and shutdown to acquire the airborne HTO dynamics. During the survey, 20 prepared passive samplers with about 30 g of zeolite 4 Å were deployed in the same locations set on the lowest layer of the reactor 3D survey. After one-day sampling, the passively collected samples were analyzed using the established analytical protocol. The HTO volumetric concentration (C, Bq m^{-3}) for each point was estimated based on the measured ^{3}H specific activity and the back-calculated humidity.

Given that the radiation regulation does not allow anyone to drink or eat inside the reactor hall, we here only consider the internal dose exposure of HTO intake by the inhalation and skin adsorption for the employees. In the hypothetical scenario, the accumulated dose by HTO (D, Sv) for an adult employee is originated from work in the reactor hall for eight hours per day for one year. It should be underscored that this scenario is a very conservative consideration from the viewpoint of radiation protection and that the actual dose exposure would be lower than our estimation. Referring to a previous work,[1] the ^{3}H intake dose to the employee could be estimated by Equation 11.

$$D = W \times DCF \times C \times R \times t \tag{11}$$

where W is the weighting coefficient considering the inhalation and skin adsorption for HTO vapor, and the value of 1.5 was adopted; DCF is the dose conversation factor for HTO, and the value of 1.8×10^{-11} Sv Bq^{-1} recommended by the International Commission on Radiological Protection

(ICRP) was used; *R* represents the breathing rate for an adult, and the value recommended by IAEA of 3.3×10^{-4} m^3 s^{-1} was considered here; *t* is the exposure time in the unit of s.

REFERENCE

(1) Nie, B.; Yang, J.; Yuan, Y.; Li, F. Additional Radiation Dose Due to Atmospheric Dispersion of Tritium Evaporated from a Hypothetical Reservoir. *Appl. Radiat. Isot.* **2021**, *167*, 109475. https://doi.org/10.1016/j.apradiso.2020.109475.

7. M&M: QA/QC

Strict quality control procedures were undertaken for both laboratory studies and field investigations. The adsorbent was pre-dried in an oven at 60 °C for 48 hours and then stored in an evacuated drying container. The equilibrium of the adsorbent was checked before its use to ensure the negligible influence of residual water in the adsorbent on the adsorption process. At each experiment batch, about 3.5 g of zeolite 4 Å (spherical 3 – 5 mm) was used for a few days' exposure at the laboratory fume hood, and the uptake was measured for equilibrium load calculation (i.e., equilibrium uptake / fresh weight). The results showed that the equilibrium load of zeolite has been relatively stable (Table S2).

Before leaching adsorbent, the ^3H level in triple distilled water was examined to avoid the influence caused by ^3H-contaminated water on the ^3H analysis. The results exhibited that the blank water is relatively stable and comparable in this study (Table S3, 1.96 ± 0.27 for Tri-Carb 2910RT and 13.03 ± 0.27 for Hidex 300SL). The sealing film (PARAFILM®) was used in a leaching beaker to avoid the evaporation of leachate from the gap between the beaker and the top lid.

The same samples were cross-analyzed and compared to test the reliability of the measured results provided by two LSC systems. In addition, a laboratory comparison with the Österreichische Agentur für Gesundheit und Ernährungssicherheit GmbH (AGES) was conducted to further examine the performance of our LSC systems. ^3H samples prepared by our laboratory and ^3H standard prepared by AGES were measured in three LSC systems, including Tri-Carb 2910RT, Hidex 300SL, and Quantulus 1220. It should be noted that the data beyond two standard deviations of average value were excluded as default in LSC analysis. The results (Figure S5 and Figure S6) demonstrate that the ^3H specific activity provided by two LSC systems are generally

comparable, and the values measured by our LSC systems also agreed well with the value given by Quantulus 1220 in the AGES.

In the field investigations, the sampler assembly was done on site where the adsorbent sealed in a beaker was transferred to the mesh cylinder to prevent additional water vapor adsorption during the preparation. Similarly, the used adsorbent was sealed in the beaker immediately after sampling to reduce additional sampling before sample preparation.

Table S2. Equilibrium load of zeolite 4 Å used in different experimental batches.

No.	Fresh weight (g)	Equilibrium weight (g)	Uptake (g)	Equilibrium load (%)[1]
1	3.56	4.32	0.76	21.35
2	3.52	4.3	0.78	22.16
3	3.49	4.26	0.77	22.06
4	3.51	4.29	0.78	22.22
5	3.52	4.29	0.77	21.88
6	3.54	4.33	0.79	22.32
7	3.54	4.33	0.79	22.32
8	3.51	4.28	0.77	21.94
9	3.52	4.29	0.77	21.88
10	3.54	4.35	0.81	22.88
Mean ± SD (k = 1)				22.1% ± 0.4%

1. Equilibrium load = Uptake/Fresh weight *100%

Table S3. Background counting rate for Tri-Carb 2910RT and Hidex 300 SL in our laboratory.

No.	LSC system	Used sample (mL)	Counting time (min)	Counting rate (cpm)
1	Tri-Carb 2910RT	10.01	840	1.91
2		10.00		2.01
3		10.02		1.93
4		10.00		1.92
5		10.00		2.07
6		10.01		1.94
Mean ± SD (k = 1)				1.96 ± 0.06
7	Hidex 300SL	10.01	840	13.19
8		10.00		13.39
9		10.02		12.69
10		10.00		13.05
11		10.00		12.90
12		10.01		12.98
Mean ± SD (k = 1)				13.03 ± 0.24

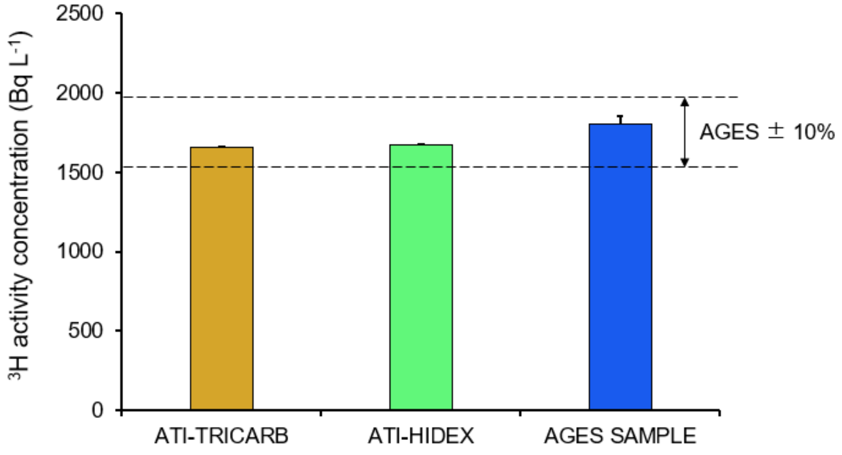

Figure S5. Cross comparison of ^{3}H measurement in different LSC systems using AGES sample. The error bars in TRIGA Center Atominstitut (ATI) samples represent the uncertainty of 24-h measurement (k = 2) and the one in AGES sample is the uncertainty of ^{3}H source. The dosh lines are the area of ±10% error of the ^{3}H specific activity referred to AGES.

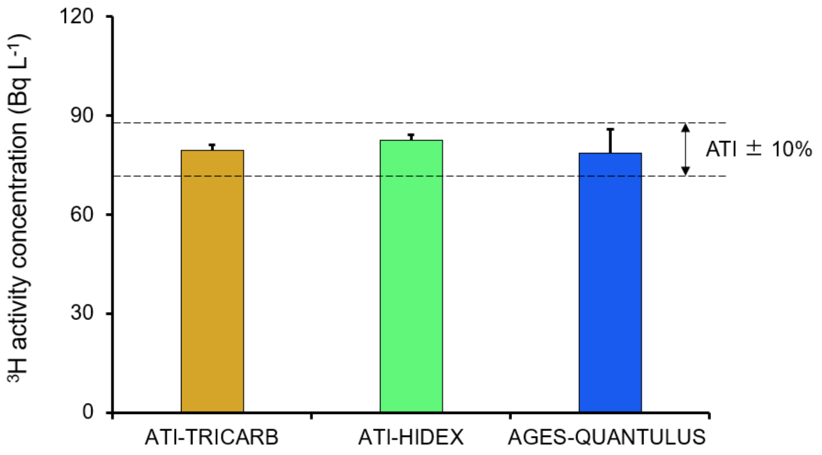

Figure S6. Cross comparison of ^{3}H measurement in different LSC systems using ATI sample. The error bars shown in ATI and AGES represent the uncertainties of 24-h measurement (k = 2) and 7-h measurement (k = 1), respectively. The dosh lines are the area of ±10% error of the ^{3}H specific activity referred to the ATI.

8. R&D: Characteristics of leachate under different conditions

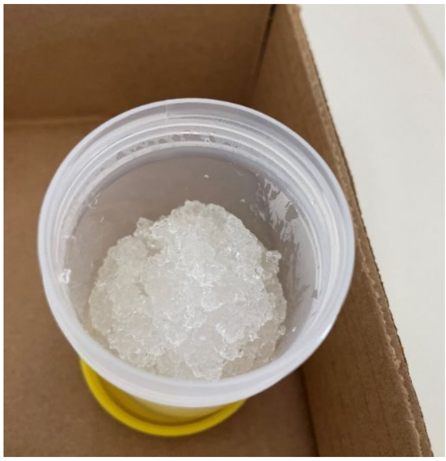

Figure S7. Strong swelling effect after adding 35 mL triple distilled water in silica gel without color indicator (B).

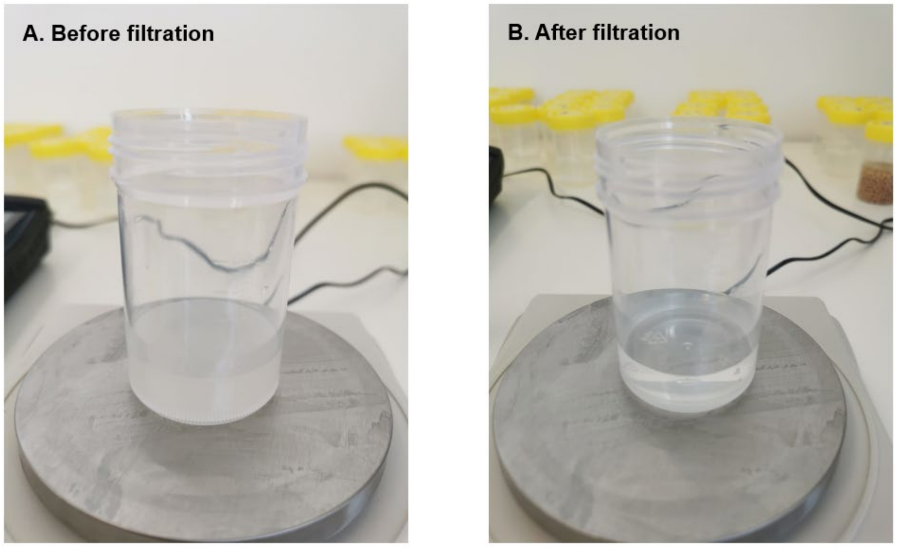

Figure S8. The transparency of leachate before (A) and after (B) using filter.

Table S4. Extracted water and conductivity in different leaching amounts.[1]

Added water (mL)	Extracted water (mL)	Filtered water (mL)	Conductivity (mS/cm)	Filtered conductivity (mS/cm)
10	0.05 ± 0.04	/	/	/
20	4.06 ± 0.09	3.59 ± 0.02	/	/
25	8.35 ± 0.08	7.98 ± 0.06	4.16 ± 0.02	4.11 ± 0.02
27	11.02 ± 0.01	10.63 ± 0.11	3.93 ± 0.01	3.95 ± 0.04
30	14.24 ± 0.19	13.15 ± 0.13	3.68 ± 0.03	3.64 ± 0.03
35	19.68 ± 0.13	19.10 ± 0.07	3.68 ± 0.02	3.55 ± 0.02
40	25.01 ± 0.07	24.68 ± 0.07	3.43 ± 0.02	3.36 ± 0.02
45	30.18 ± 0.14	26.69 ± 0.12	3.18 ± 0.01	3.12 ± 0.01
50	35.15 ± 0.08	34.70 ± 0.05	2.98 ± 0.01	2.93 ± 0.01

1. Results are presented as mean ± SD (k =1). Three replicates samples were used.

9. R&D: Flow chart of the optimized leaching method

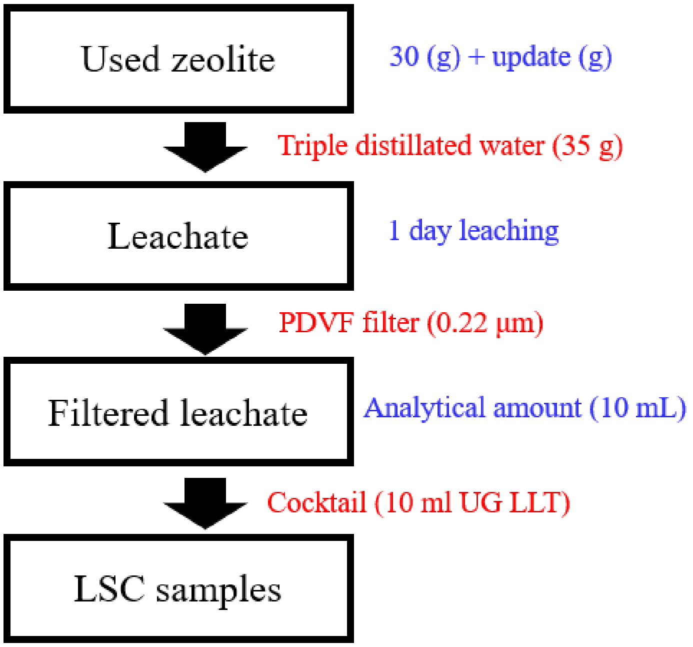

Figure S9. Flow chart of the optimized leaching method.

10. R&D: Radiometric properties of leaching method

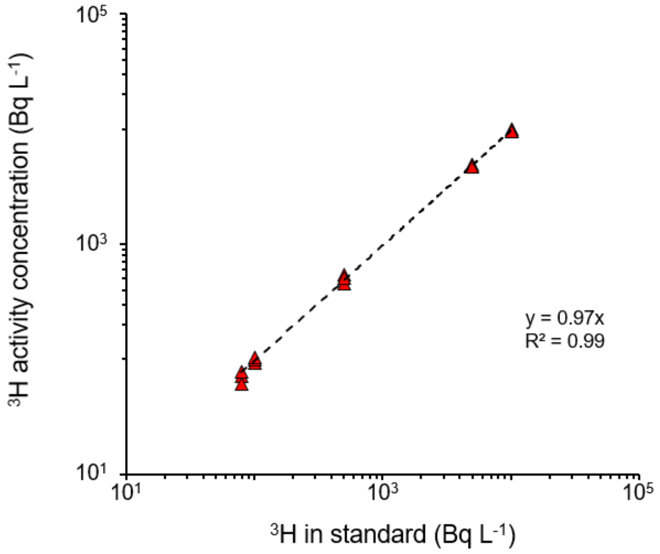

Figure S10. The linear relationship between the ^{3}H specific activity in the leachate and the spiked ^{3}H of the standard. Each concentration point includes three replicate samples.

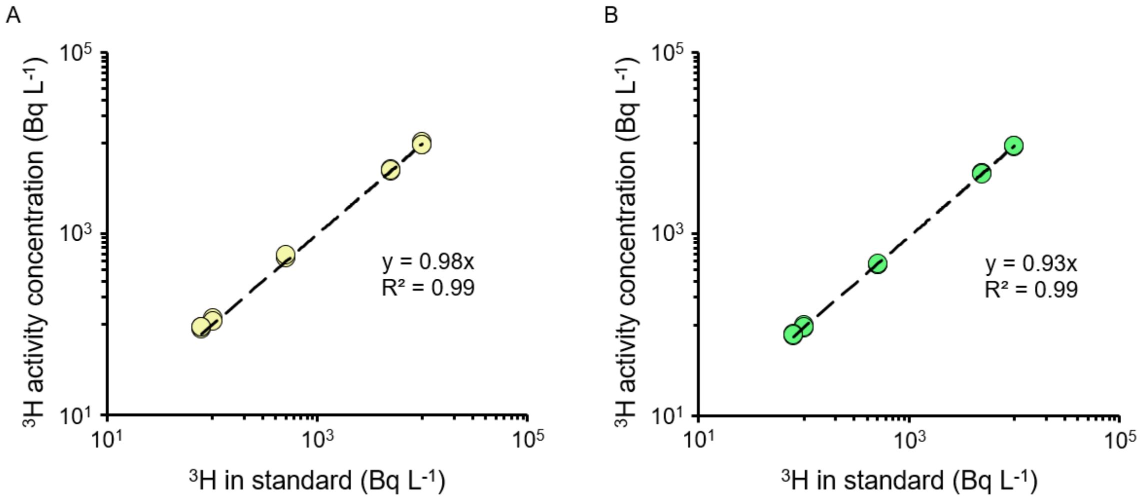

Figure S11. The linear relationship between the ^{3}H specific activity in the leachate and the spiked ^{3}H of the standard after one month (A) and four months (B) of preparation. Each concentration point includes three replicate samples.

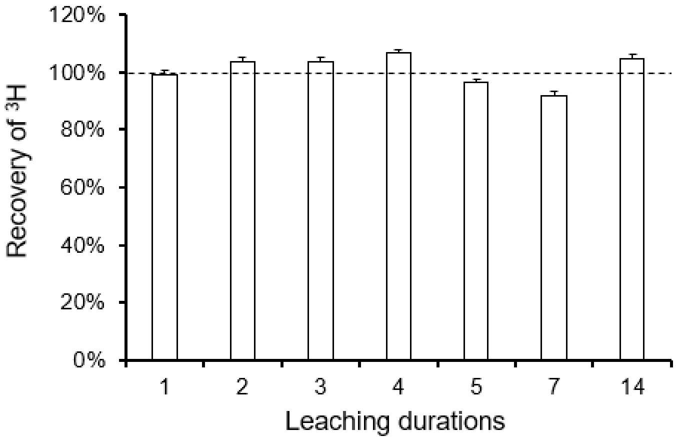

Figure S12. Recovery of ^{3}H under different leaching durations. the dotted line is the recovery of 100%, and the error bar indicates the combined uncertainty (k = 1).

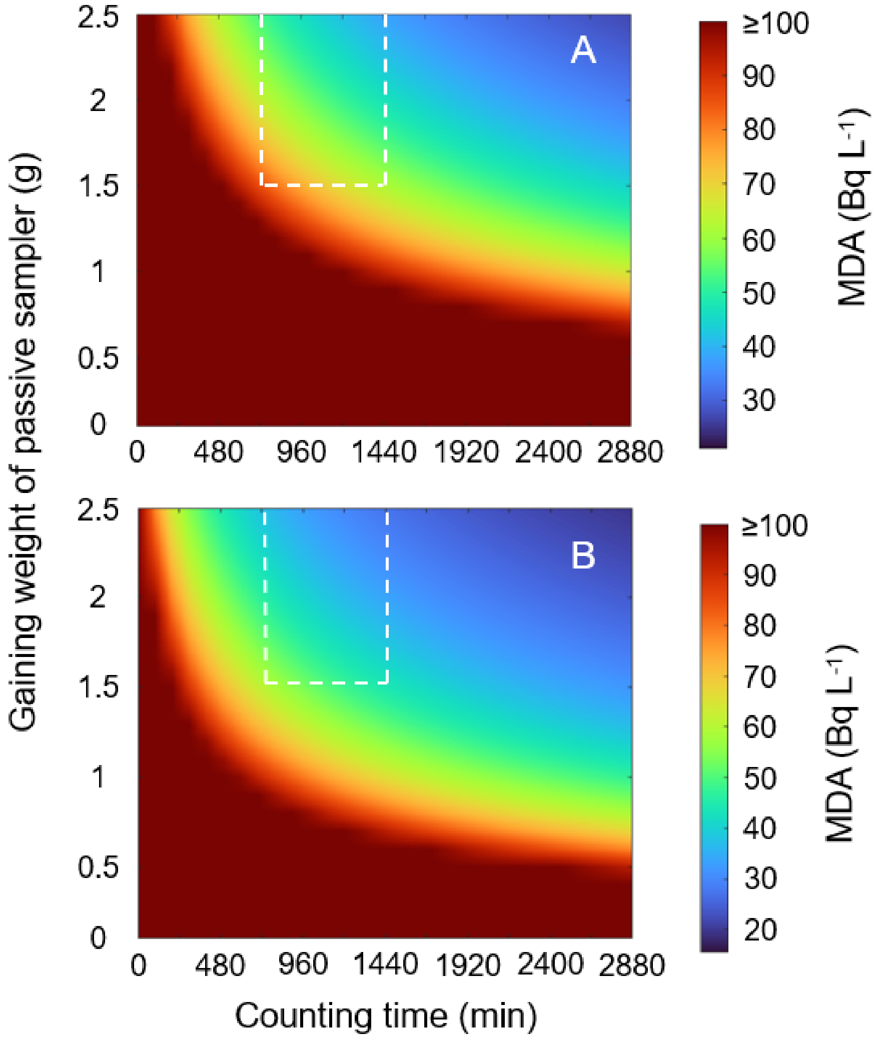

Figure S13. The method's MDA in Hidex 300SL (A) and Tri-Carb 2910RT (B), respectively. The white dotted box is the MDA in the ideal region where gaining weight falls in 1.5 to 2.5 g, and the counting time is about 6 – 24 h.

11. R&D: Reactor investigation

Table S5. Detailed information on airborne HTO in the reactor hall.

No.	Uptake (g)	^3H specific activity ± uncertainty (Bq L^{-1})	HTO volumetric concentration ± uncertainty (mBq m^{-3})	Note
1	1.88	23.84 ± 23.84	85.12 ± 85.12	<MDA
2	3.26	14.23 ± 14.23	103.26 ± 103.26	<MDA
3	1.28	34.42 ± 34.42	79.05 ± 79.05	<MDA
4	1.41	31.34 ± 31.34	80.24 ± 80.24	<MDA
5	0.79	55.04 ± 55.04	74.74 ± 74.74	<MDA
6	1.63	27.34 ± 27.34	82.59 ± 82.59	<MDA
7	1.72	25.98 ± 25.98	83.54 ± 83.54	<MDA
8	1.64	27.23 ± 27.23	82.85 ± 82.85	<MDA
9	1.58	28.22 ± 28.22	82.26 ± 82.26	<MDA
10	1.88	23.85 ± 23.85	85.17 ± 85.17	<MDA
11	1.66	26.89 ± 26.89	82.96 ± 82.96	<MDA
12	1.76	25.48 ± 25.48	84.18 ± 84.18	<MDA
13	2.04	22.05 ± 22.05	86.87 ± 86.87	<MDA
14	2.09	21.56 ± 21.56	87.43 ± 87.43	<MDA
15	0.91	48.03 ± 48.03	75.90 ± 75.90	<MDA
16	1.25	35.37 ± 35.37	79.10 ± 79.10	<MDA
17	1.45	30.55 ± 30.55	80.71 ± 80.71	<MDA
18	2.1	21.45 ± 21.45	87.52 ± 87.52	<MDA
19	1.95	22.98 ± 22.98	85.72 ± 85.72	<MDA
20	1.89	57.72 ± 15.40	207.42 ± 55.33	
21	1.26	35.11 ± 35.11	79.23 ± 79.23	<MDA
22	1.27	34.74 ± 34.74	79.09 ± 79.09	<MDA
23	1.15	38.19 ± 38.19	77.87 ± 77.87	<MDA
24	1.85	24.27 ± 24.27	85.03 ± 85.03	<MDA
25	2.79	16.49 ± 16.49	96.55 ± 96.55	<MDA

Continued Table S4

No.	Uptake (g)	^{3}H specific activity ± uncertainty (Bq L^{-1})	HTO volumetric concentration ± uncertainty (mBq m^{-3})	Note
26	1.12	39.29 ± 39.29	77.82 ± 77.82	<MDA
27	1.2	36.68 ± 36.68	78.39 ± 78.39	<MDA
28	1.3	33.96 ± 33.96	79.34 ± 79.34	<MDA
29	1.98	22.70 ± 22.70	86.26 ± 86.26	<MDA
30	1.34	33.08 ± 33.08	79.96 ± 79.96	<MDA
31	1.38	32.04 ± 32.04	80.06 ± 80.06	<MDA
32	1.31	33.82 ± 33.82	79.70 ± 79.70	<MDA
33	1.91	23.53 ± 23.53	85.63 ± 85.63	<MDA
34	1.18	37.33 ± 37.33	78.32 ± 78.32	<MDA
35	0.77	56.66 ± 56.66	74.86 ± 74.86	<MDA
36	1.29	34.19 ± 34.19	79.21 ± 79.21	<MDA
37	1.41	31.38 ± 31.38	80.33 ± 80.33	<MDA
38	1.26	35.09 ± 35.09	79.17 ± 79.17	<MDA
39	1.26	35.10 ± 35.10	79.21 ± 79.21	<MDA
40	0.74	58.94 ± 58.94	74.65 ± 74.65	<MDA
41	1.14	38.53 ± 38.53	77.83 ± 77.83	<MDA
42	1.29	34.21 ± 34.21	79.25 ± 79.25	<MDA
43	2.12	85.83 ± 13.69	354.19 ± 56.51	
44	1.49	104.70 ± 19.60	285.33 ± 53.42	
45	1.76	82.89 ± 16.67	273.83 ± 55.08	
46	1.16	38.00 ± 38.00	78.22 ± 78.22	<MDA
47	1.07	41.03 ± 41.03	77.30 ± 77.30	<MDA
48	1.67	26.74 ± 26.74	83.09 ± 83.09	<MDA
49	1.35	32.75 ± 32.75	79.82 ± 79.82	<MDA
50	1.71	115.71 ± 17.56	369.57 ± 56.08	
51	1.14	38.51 ± 38.51	77.78 ± 77.78	<MDA
52	1.22	36.08 ± 36.08	78.54 ± 78.54	<MDA
53	2.09	21.64 ± 21.64	87.76 ± 87.76	<MDA

Continued Table S4

No.	Uptake (g)	^{3}H specific activity ± uncertainty (Bq L^{-1})	HTO volumetric concentration ± uncertainty (mBq m^{-3})	Note
54	1.72	25.97 ± 25.97	83.52 ± 83.52	<MDA
55	0.75	58.04 ± 58.04	74.57 ± 74.57	<MDA
56	1.12	39.19 ± 39.19	77.62 ± 77.62	<MDA
57	1.23	35.85 ± 35.85	78.76 ± 78.76	<MDA
58	1.27	34.77 ± 34.77	79.15 ± 79.15	<MDA
59	1.46	30.34 ± 30.34	80.81 ± 80.81	<MDA
60	0.77	241.28 ± 38.11	318.79 ± 50.35	
61	1.24	35.55 ± 35.55	78.81 ± 78.81	<MDA
62	0.85	209.93 ± 33.91	308.26 ± 49.79	
63	1.22	36.08 ± 36.08	78.54 ± 78.54	<MDA
64	1.03	42.51 ± 42.51	76.82 ± 76.82	<MDA
65	1.2	109.41 ± 24.15	233.84 ± 51.63	
66	1.09	40.35 ± 40.35	77.57 ± 77.57	<MDA
67	1.1	83.88 ± 26.28	162.88 ± 51.04	
68	1.12	39.22 ± 39.22	77.69 ± 77.69	<MDA
69	2.25	72.62 ± 13.29	322.47 ± 59.05	
70	2.32	50.89 ± 13.02	234.76 ± 60.07	
71	1.2	36.71 ± 36.71	78.46 ± 78.46	<MDA
72	1.17	37.56 ± 37.56	78.06 ± 78.06	<MDA
73	1.32	33.50 ± 33.50	79.62 ± 79.62	<MDA
74	1.02	195.55 ± 28.73	349.65 ± 51.38	
75	1.56	28.57 ± 28.57	82.06 ± 82.06	<MDA
76	1.68	68.65 ± 17.42	214.81 ± 54.51	
77	2.1	21.54 ± 21.54	87.88 ± 87.88	<MDA
78	1.63	27.46 ± 27.46	82.97 ± 82.97	<MDA
79	1.63	67.74 ± 18.30	204.65 ± 55.29	
80	0.98	44.81 ± 44.81	76.72 ± 76.72	<MDA
81	1.39	199.43 ± 21.68	502.31 ± 54.60	

Continued Table S4

No.	Uptake (g)	^{3}H specific activity ± uncertainty (Bq L^{-1})	HTO volumetric concentration ± uncertainty (mBq m^{-3})	Note
82	1.54	161.54 ± 18.83	457.13 ± 53.29	
83	1.59	165.30 ± 18.73	485.26 ± 55.00	
84	1.59	160.97 ± 18.79	472.55 ± 55.16	
85	0.98	448.78 ± 31.04	768.28 ± 53.15	
86	1.39	375.26 ± 21.69	945.19 ± 54.65	
87	1.46	234.28 ± 20.54	623.84 ± 54.71	
88	1.05	165.74 ± 27.74	305.87 ± 51.21	
89	1.22	203.92 ± 24.20	443.89 ± 52.68	
90	1.07	239.99 ± 28.25	452.12 ± 53.23	
91	1.2	223.84 ± 25.06	478.41 ± 53.57	
92	1.14	205.58 ± 25.78	415.18 ± 52.06	
93	0.95	184.32 ± 30.52	305.09 ± 50.52	
94	1.03	456.48 ± 29.15	824.91 ± 52.68	
95	1.07	384.11 ± 27.91	723.62 ± 52.59	
R1	0.77	471.16±38.83	626.46 ± 49.67	
R2	0.72	618.73±42.05	761.20 ± 50.09	
R3	0.65	423.86±45.64	468.04 ± 48.80	

2

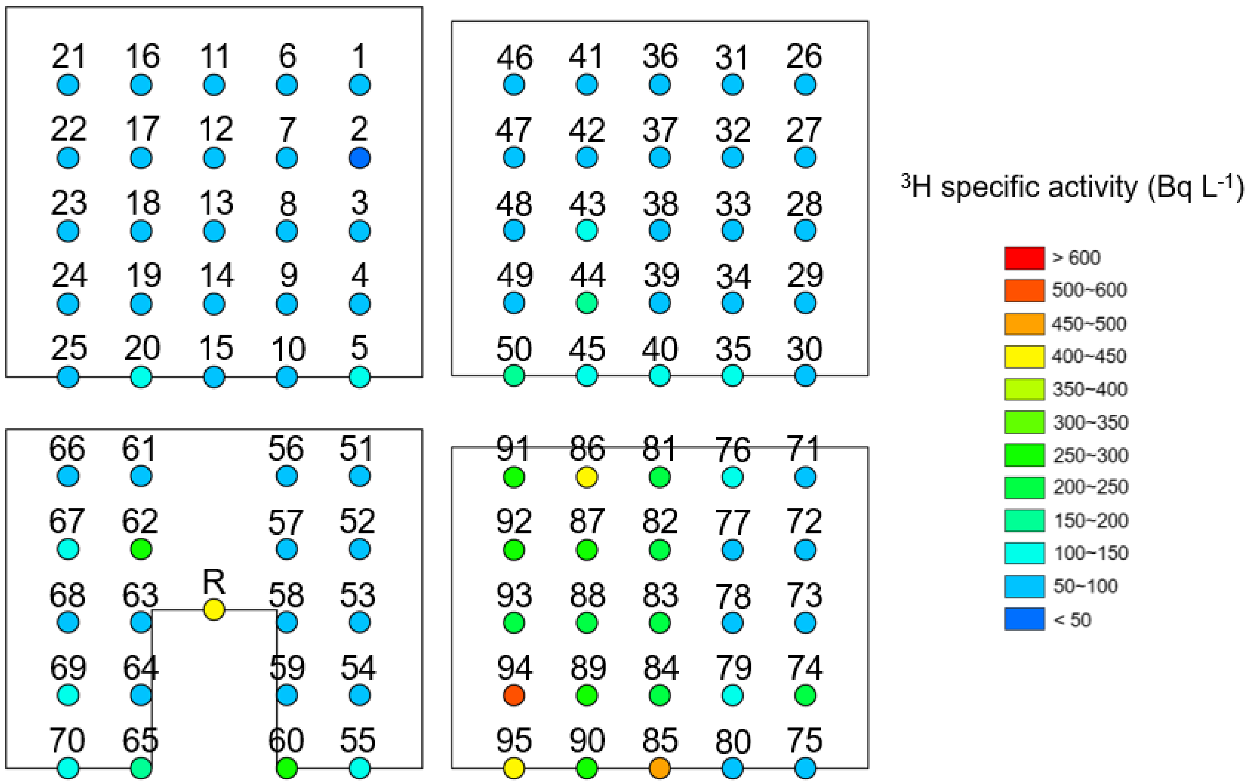

Figure S14. Spatial distribution of HTO specific activity in different vertical profiles. The value used in the reactor (i.e., R) is weighted average by the humidity of three samples.

12. R&D: Spatial profiles of ^{3}H and dose rate in groundwork area

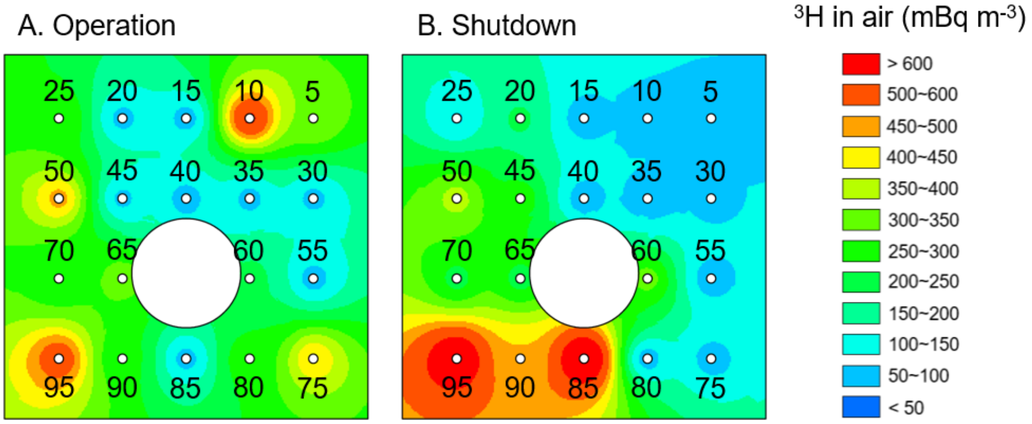

Figure S15. Spatial distribution of airborne HTO concentration in the ground-floor work area at operation (A) and shutdown (B) stages.

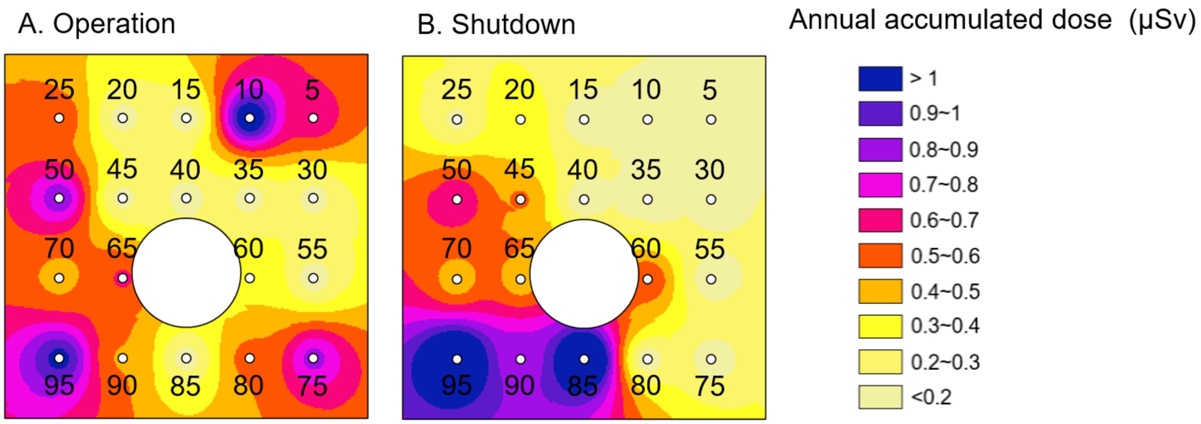

Figure S16. Spatial distribution of annual accumulated dose in the ground-floor work area at operation (A) and shutdown (B) stages.
